# Effects of glucose availability in *Lactobacillus sakei*; metabolic change and regulation of the proteome and transcriptome

**DOI:** 10.1371/journal.pone.0187542

**Published:** 2017-11-03

**Authors:** Anette McLeod, Ellen F. Mosleth, Ida Rud, Filipe Branco dos Santos, Lars Snipen, Kristian Hovde Liland, Lars Axelsson

**Affiliations:** 1 Nofima AS, Norwegian Institute of Food, Fisheries and Aquaculture Research, Ås, Norway; 2 Molecular Microbial Physiology Group, Swammerdam Institute for Life Sciences, Faculty of Science, University of Amsterdam, Amsterdam, The Netherlands; 3 Department of Chemistry, Biotechnology and Food Sciences, Norwegian University of Life Sciences, Ås, Norway; University of Torino, ITALY

## Abstract

Effects of glucose availability were investigated in *Lactobacillus sakei* strains 23K and LS25 cultivated in anaerobic, glucose-limited chemostats set at high (*D* = 0.357 h^-1^) and low (*D* = 0.045 h^-1^) dilution rates. We observed for both strains a shift from homolactic towards more mixed acid fermentation when comparing high to low growth rates. However, this change was more pronounced for LS25 than for 23K, where dominating products were lactate>formate>acetate≥ethanol at both conditions. A multivariate approach was used for analyzing proteome and transcriptome data from the bacterial cultures, where the predictive power of the omics data was used for identifying features that can explain the differences in the end-product profiles. We show that the different degree of response to the same energy restriction revealed interesting strain specific regulation. An elevated formate production level during slow growth, more for LS25 than for 23K, was clearly reflected in correlating pyruvate formate lyase expression. With stronger effect for LS25, differential expression of the Rex transcriptional regulator and NADH oxidase, a target of Rex, indicated that maintainance of the cell redox balance, in terms of the NADH/NAD^+^ ratio, may be a key process during the metabolic change. The results provide a better understanding of different strategies that cells may deploy in response to changes in substrate availability.

## Introduction

A superior performance of the lactic acid bacterium (LAB) *Lactobacillus sakei* in meat fermentation has been explained by its genomic and physiological adaptions to the raw-meat environment [1]. The bacterium is useful in industrial meat fermentation, where important attributes for starter cultures are effective growth and high acidification activity. *L*. *sakei* is strictly fermentative, completely devoid of a respiratory chain, however, still aerotolerant [1, 2]. Depending on the growth condition, the bacterium can shift between a pathway leading to nearly exclusively lactate production versus a pathway leading to the production of mixed acids. In general, homofermentative LAB convert carbohydrates into lactate using the Embden-Meyerhof pathway (EMP). As a bacterial substrate, meat contains a restricted amount and diversity of carbohydrates, mostly consisting of low-levels of glucose derived from glycogen. Therefore, exogenous glucose is often added to speed up and improve the ripening process of fermented meat products. Hexose fermentation in *L*. *sakei* is homolactic and proceeds via EMP, resulting in formation of lactic acid and subsequent decrease in pH [1–3]. Heterofermentative LAB use the phosphoketolase pathway (PKP), which is often used by LAB to ferment pentoses [4]. In meat, ribose is also available, mostly derived from ATP hydrolysis, and is fermented via the heterolactic PKP in *L*. *sakei* [2, 3]. Reflecting the ability to metabolize meat components such as proteins, *L*. *sakei* has a comprehensive set of transporters [1, 5, 6]. An efficient utilization of nucleosides closely linked with the uptake and catabolic machinery of ribose [1, 3, 7–9], and conversion of certain amino acids such as arginine through the arginine deiminase (ADI) pathway [10, 11], provides this species with additional energy sources and thus competitive advantages in the meat environment.

The study of bacteria at a defined growth rate can be achieved in the chemostat [12], in which the specific growth rate is directly manipulated by changing the dilution rate, creating a controlled and constant environment, so-called steady-state. In the present work, we grew two strains of *L*. *sakei* in anaerobic, glucose-limited chemostats at two different growth rates, where fast and slow growth demonstrate adaption to glucose availability, which in many organisms results in a shift in metabolic strategy that generates less ATP per glucose in situations with high glucose availability [13–16]. We chose to study *L*. *sakei* strain 23K, originally isolated from sausage [17], and the commercial starter culture strain LS25 from fermented sausage [18] for three reasons. First, both strains have publicly available genome sequences [1, 19]. Second, the strains represent the two different *L*. *sakei* subspecies, *carnosus* and *sakei*, respectively [20–22]. Third, they differ in their patterns of growth and in metabolic/fermenting capacities [8, 21, 23]. LS25 has been shown to ferment a larger variety of carbohydrates than 23K and to grow faster on ribose. Also, LS25 seems to be a faster acid producer which shows better abilities as a starter culture based on acidification properties in a meat model and its ability to compete with the indigenous microbiota of meat batter, compared with 23K. We here investigate and compare how growth rate-dependent relative protein and gene transcript levels relate to the metabolic end-product formation profiles of the two *L*. *sakei* strains. The main goal was to unravel underlying molecular mechanisms involved in the response to reduced glucose availability.

## Materials and methods

### Bacterial strains and growth medium

*L*. *sakei* strain 23K is a plasmid-cured sausage isolate [17], and strain LS25 is a commercial starter culture strain for salami sausage [18]. The complete and a draft genome sequence have been published for 23K [1] and for LS25 [19], respectively. Both strains were grown in a chemically defined medium (CDM) which is specifically designed to support the growth of LAB [24, 25]. The viability of cells from the chemostat cultures was determined by conventional plating on agar plates of the rich De Man, Rogosa and Sharpe (MRS) medium (Oxoid) and grown at 30°C for 48 h.

### Chemostat cultures

Anaerobic glucose-limited chemostat cultures were grown in 300-ml fermenter vessels (Soham Scientific) with a total volume of 280 ml culture under continuous stirring. The temperature was kept at 30°C and the pH kept constant at pH 6.5 by automatic addition of 2 M NaOH. Growth rates of the steady-states were controlled by the culture dilution rates (*D*) set at 0.045 h^-1^ (henceforth also referred to as low growth rate) and 0.357 h^-1^ (high growth rate). The inoculum for each chemostat culture was prepared through two 10-mL subcultures for 16 h each, with a transfer volume of 5% (vol vol^-1^). After batch growth until an optical density at 600 nm (OD_600_) of around 2 was reached, fresh medium was pumped at the appropriate flow. The cultures were considered to be in steady-state since no detectable glucose remained in the culture supernatant and the ODs, dry weights, and product concentrations of the cultures were constant over two consecutive days. All experiments were performed in triplicate, resulting in sets of 12 samples (2 strains, 2 growth rates, 3 biological replicates) for further analyses.

### Sampling

The chemostat cultures were grown for six generations before sampling. Biomass was monitored by measuring the OD_600_, and biomass dry weight was determined gravimetrically. For proteomic analysis, cells were harvested by centrifugation at 2800 x *g* at 4°C and washed twice in 0.01 M Tris-HCl pH 7.5 for 15 min before storage at -80°C. Supernatants were frozen at -20°C until metabolite analysis. For transcriptomic analysis, samples (3 ml) were collected and added RNA Protect Bacteria Reagent (Qiagen) (6 ml), vortexed for 5 sec, incubated at room temperature for 5 min, centrifuged for 10 min at 3700 x *g*, before the pellet was stored at -80°C for later RNA purification.

### Metabolite analysis

**S**upernatant samples were filtered through a 0.22 μm Millex-HV Durapore PVDF filter (Millipore), and extracellular concentrations of glucose, pyruvate, lactate, formate, acetate, succinate, acetoin, butanediol and ethanol were analyzed on an Agilent 1100 series high-pressure liquid chromatography (HPLC) system (Agilent Technologies) equipped with an auto sampler cooled to 4°C, a diode array detector (DAD), and a RI 132 refractive index (Gilson) detector. A Rezex organic acid analysis column (Phenomenex) at a temperature of 45°C with 7.2 mM H_2_SO_4_ as the eluent was used as previously described [24]. ChemStation chromatography software (Agilent) was used for data integration, and concentrations were estimated by comparison of peak areas to a calibration curve obtained with standards analyzed under the same conditions.

The extracellular concentration in the filtered supernatants of alanine, arginine, aspartic acid, asparagine, citrulline, glutamic acid, glutamine, glycine, histidine, isoleucine, leucine, lysine, methionine, ornithine, phenylalanine, serine, threonine, tryptophan, tyrosine, and valine were determined by liquid chromatography with fluorescence detection (350 nm excitation, 450 nm emission, RF 20-A, Shimadzu, ‘s-Hertogenbosch, The Netherlands) as detailed elsewhere [26]. The method is based on a precolumn derivatization protocol with ortho-phtalaldehyde and 3-mercaptopropionic acid, using 1 mM of DL-norvaline as an internal standard. Compounds listed were quantified by comparison of peak areas against normalized individual calibration lines obtained under the same conditions.

### Protein extraction and NanoLC-ESI-LTQ Orbitrap Velos Pro mass spectrometry analysis

Extraction of soluble proteins from 10 ml samples of the cultures was performed as previously described by McLeod *et al*. (8). The protein concentration of the cytosolic fraction was determined using the colorimetric assay *RC DC* Protein Assay (Bio-Rad), with bovine serum albumin (BSA) as standard protein, according to the manufacturer’s instructions. Trypsin digested samples (~0.5 μg) were loaded in randomized order onto a pre-column (Dionex, Acclaim PepMap Nano Trap column, C18, 75 μm i.d. x 2 cm, 3 μm) followed by separation on an analytical column (Dionex, Acclaim PepMap100 RSLCnano column, 75 μm x 15 cm, C18, 2 μm) using a Dionex Ultimate NCS-3500RS LC system (Sunnyvale, CA) coupled online to an Orbitrap Velos Pro (Thermo Scientific) mass spectrometer (MS). A linear gradient of 90 minutes was used with mobile phase A (0.1% formic acid / 2% acetonitrile) and mobile phase B (0.1% formic acid / 90% acetonitrile) ramping from 8–38% mobile phase B, and the peptides were continuously eluted into the Orbitrap MS. Data dependent acquisition was used in collision-induced dissociation-mode, where the MS continuously sequenced the 7 ions with highest intensity eluting from the column during the gradient. The raw spectra from the Orbitrap were loaded into Progenesis LC-MS v4.0 (http://www.nonlinear.com/products/progenesis/lc-ms/overview/), and retention time aligned to correlate precursor and potential fragment ion spectra across samples. Spectra were centroided, deisotoped and charge-state-reduced to produce a single accurately mass measured monoisotopic mass for each peptide and the associated fragment ions. The data were combined in a merge file and searched against *L*. *sakei* using SeachGUI [27]. The search was open in PeptideShaker (https://code.google.com/p/peptide-shaker/) and the identified proteins were transported back to Progenesis to fulfill the labelfree analysis (peptide FDR = 1.0%). Normalized abundance values followed by log_2_ transformation were used as inputs in statistical analyses.

### RNA isolation, mRNA enrichment, cDNA synthesis and RNA sequencing

Total RNA was extracted from bacterial cells using the RNeasy Mini Kit (Qiagen) as described by Rud *et al*. [28]. The RNA was quantified spectrophotometrically (NanoDrop ND-1000; NanoDrop Technologies), and quality and purity were verified using an Agilent 2100 Bioanalyzer (Agilent Technologies). Sample criteria were A260/A280 ratio >1.9; A260/A280 ratio ∼2.0; 23S/16S RNA ratio >1.3; RIN>8.0 for use in the transcriptome analysis. The MICROBExpress™ Bacterial mRNA Enrichment Kit (Ambion, Life Technologies) was used for the selective removal of 16S and 23S rRNA with oligonucleotide probes attached to magnetic beads according to the manufacturer’s protocol. Furthermore, enriched mRNA yields were analyzed using NanoDrop ND-1000 and Agilent 2100 Bioanalyzer, before generation of cDNA sequencing libraries with the Illumina TruSeq RNA Sample Prep Kit following instructions from the supplier (Illumina Netherlands BV, #RS-122-2001). The mRNA is by this procedure fragmented into small pieces using divalent cations under elevated temperature, copied into first strand cDNA using SuperScript® II Reverse Transcriptase and random primers (Invitrogen). This is followed by second strand cDNA synthesis using DNA polymerase I and RNase H. The cDNA fragments then go through an end repair process, the addition of a single ‘A’ base, and then ligation of RNA adapters index (barcoding). The products are purified and enriched with PCR to create the final cDNA library. Agencourt AMPure XP (Beckman Coulter, #A63880) solid-phase paramagnetic bead technology was used for the purification of PCR amplicons. Double-stranded cDNA was quantified and the quality and purity were verified using the NanoDrop ND-1000 and the Agilent 2100 Bioanalyzer, respectively. The cDNA libraries were sequenced (50 bp read length, 12-plex, one lane) on a HiSeq system (Illumina). The raw reads have been deposited to the National Center for Biotechnology Information (NCBI) Sequence Read Archive (SRA) under accession number PRJNA393246.

### RNA sequencing-based transcriptome data

First, the software bowtie [29] was used to construct a reference database from each of the two strains in the study, consisting of 1963 and 2027 annotated genes for *L*. *sakei* strain 23K [1] and strain LS25 [19], respectively. Next, reads in fastq formatted files were mapped to the corresponding reference database using the command line “bowtie -n 2—trim5 10 –best <filenames>”, which means we allow up to 2 mismatches and trim each read with 10 nt in the 5-end. The latter was due to a quality inspection of the reads using the FastQC software (http://www.bioinformatics.babraham.ac.uk/projects/fastqc/). The counts for each strain were arranged in a matrix of N rows (N = 1963 for 23K, N = 2027 for LS25) and 6 columns, where 3 of the columns are data from low and 3 from high growth rate conditions, using the software R (http://www.r-project.org/). The 16S and 23S rRNA-genes usually consume the majority of the sequencing depth in microbial RNAseq samples, and the total number of reads mapped in each sample is very sensitive to these genes. Despite selective removal of the 16S and 23S rRNA using the MICROBExpress™ Bacterial mRNA Enrichment Kit, these loci still influence largely and were therefore discarded from the data set. The read counts were converted to RPKM-values (Reads Per Kilobase gene per Million mapped reads) in the Nx6 matrices described above, followed by log_2_ transformation before being used as inputs in statistical analyses.

### Data exploration and statistical analyses

Effects of strains and growth condition for the phenotypic end-product profiles (henceforth also referred to as phenome) were defined by F-test in analysis of variance for lactate, formate, ethanol and acetate after adjusting p-value by False Discovery Rate (FDR) using the rotation test described by Langsrud *et al*. [30]. Similar approach was used to define effects of strains and growth condition for the amino acids. The tests were performed in R using the package ffmanova (https://CRAN.R-project.org/package=ffmanova). For the proteome (abundance values, log_2_ transformed) and transcriptome (RPKM-values, log_2_ transformed), with focus on common genes for both strains, predictions of the end-product phenotype were performed with variable selection by the multivariate elastic net method [31] using the glmnet package in R (https://CRAN.R-project.org/package=glmnet). Elastic net combines the merits of ridge regression (known also as Tikhonov regularization) and the Lasso method [32, 33]. First, the ridge regression coefficients for all variables multiplying the estimated coefficients are found, and second, a Lasso type shrinkage is performed. The method was repeated 1000 times using alpha tuning parameter 0.5, which gives a 50:50 weight to ridge regression and Lasso. The regularization parameter (lambda) was set to log.lambda.min. An explorative multivariate Principal Component Analysis (PCA) was performed within each data block (phenome, transcriptome and proteome) before and after elastic net feature extraction. To omit variables that only describe variation within the biological replicates, proteins and transcripts selected for the prediction of the phenotype were subjected to validation by univariate confidence intervals [34]. This was performed on the fold change difference within each strain.

## Results

### Metabolite analyses revealed phenotypic strain differences

**Fig 1:**
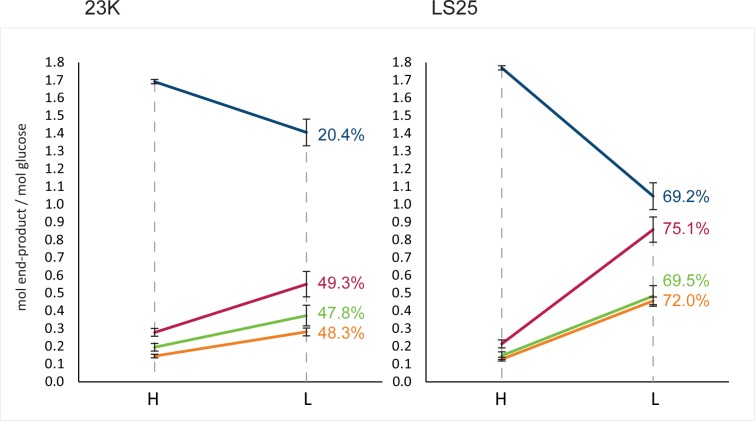
*L*. *sakei* end-product formation at high and low glucose availability. Mol end-product (lactate; blue, formate; red, acetate; green, ethanol; orange) per mol glucose for *L*. *sakei* strains 23K and LS25 grown at high (*D* = 0.357 h^-1^) and low (*D =* 0.045 h^-1^) dilution rates in continuous glucose-limited chemostats. Percent (%) change in mol of end-product per mol of glucose between the growth rates is indicated.

**Fig 2:**
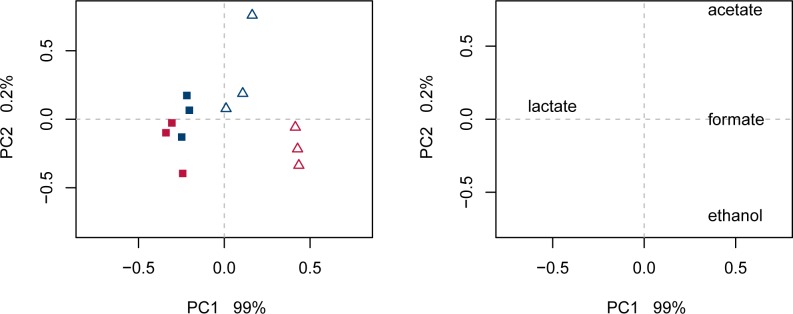
PCA on the phenome. PCA on the phenome end-products mean centered and standardized to unit variance. Score plot (left) and loading plot (right) on PC1 (x-axis) vs PC2 (y-axis). *L*. *sakei* strains 23K and LS25 are shown in blue and red, respectively. Squares indicate high growth rate and high glucose availability. Open triangles indicate low growth rate and restricted glucose availability.

For the two strains of *L*. *sakei*, 23K and LS25, cultivated under anaerobic conditions in glucose-limited chemostat cultures set at high (*D* = 0.357 h^-1^) and low (*D* = 0.045 h^-1^) dilution rates, glucose was completely consumed throughout cultivation. All chemostat results showed a carbon balance above 95% on the basis of glucose consumption and organic acid formation (Table 1). The reduction in glucose availability from high to low growth rate resulted in a significantly changed end-product profile (phenome) for both strains (S1 Table), though the dominating products were lactate>formate>acetate≥ethanol at both conditions (Fig 1). The shifts in metabolism is illustrated with PCA for mean centred standardised data (Fig 2). PC1 captured as much as 99% of the total variation in the data set, separating the samples from high vs low growth rate in the score plot (Fig 2, left), clearly more pronounced for strain LS25 than for 23K (S1 Table), and the correlating end-products in the loading plot (Fig 2, right). High growth rate correlated with lactate, reflecting homolactic fermentation, while low growth rate correlated with aceate, formate and ethanol, reflecting a more mixed acid profile. The lactate flux decreased stronger for LS25 compared with 23K from high to low growth rate (Table 1), and the end-product formations (mol per mol glucose) illustrated the highest relative change for formate for LS25, which increased 75.1% compared to 49.3% in 23K (Fig 1). The biomass concentration decreased from high to low growth rate for both strains. The dry weight decreased by 31.15% and 29.21% (Table 1), and the OD_600_ by 24.1% and 17.1% for 23K and LS25, respectively. We did not observe any significant differences in the percentage of viable cells within these populations (data not shown).

**Table 1:** Metabolite production, carbon flux and carbon flux/growth rate in *L*. *sakei* during continuous cultivation in glucose-limited CDM-LAB medium.

Strain	Dilution rate, h^-1^	Dry Weight (dW) g/liter^a^		Lactate^a^	Formate^a^	Acetate^a^	Ethanol^a^	% Carbon balance^a^^,^ ^b^
23K	0.357	1.83 (0.01)	Conc (mM)	91.78 (0.63)	15.13 (1.22)	10.57 (1.15)	7.91 (0.56)	98.32 (0.65)
			Flux (mmol h^-1^) dW^-1^	17.84 (0.12)	2.94 (0.24)	2.06 (0.22)	1.54 (0.11)	
			mmol g^-1^	49.96 (0.34)	8.24 (0.66)	5.76 (0.62)	4.30 (0.30)	
	0.045	1.26 (0.09)	Conc (mM)	76.25 (4.09)	29.85 (3.88)	20.25 (3.15)	15.28 (1.19)	
			Flux (mmol h^-1^) dW^-1^	2.72 (0.15)	1.06 (0.14)	0.72 (0.11)	0.54 (0.04)	99.00 (0.56)
			mmol g^-1^	60.34 (3.24)	23.62 (3.07)	16.03 (2.49)	12.09 (0.94)	
LS25	0.357	1.78 (0.04)	Conc (mM)	96.00 (1.79)	11.61 (1.78)	8.01 (1.03)	6.93 (2.36)	
			Flux (mmol h^-1^) dW^-1^	19.23 (0.36)	2.33 (0.36)	1.61 (0.21)	1.39 (0.47)	98.95 (1.31)
			mmol g^-1^	53.88 (1.00)	6.52 (1.00)	4.50 (0.58)	3.89 (1.32)	
	0.045	1.26 (0.07)	Conc (mM)	56.75 (0.47)	46.55 (0.20)	26.27 (0.14)	24.77 (0.89)	
			Flux (mmol h^-1^) dW^-1^	2.01 (0.02)	1.65 (0.01)	0.93 (0.01)	0.88 (0.03)	95.75 (0.37)
			mmol g^-1^	44.75 (0.37)	36.70 (0.15)	20.71 (0.11)	19.53 (0.70)	

^a^Standard deviation is shown in parentheses

^b^The CDM-LAB medium contained 55.5 mM glucose

Differences in consumption of certain amino acids were observed (S1 and S2 Tables). Alanine, lysine, and glutamine consumption decreased at low compared with high growth rate, however the changes were minor for the latter two amino acids. A simultanous increase was seen in threonine and tryptophan consumption, which was minor for the latter amino acid. The only amino acid for which the two strains responded differently upon changed growth condition, was threonine. 23K cells consumed more than 95% of threonine at both growth rates, whereas LS25 consumed approximately 73% during fast and 95% during slow growth. Strain differences regardless of growth rate was also observed. 23K consumed most of the arginine present in the growth medium, more than 93% at both growth rates, whereas LS25 consumed less than 52%. Parallel to the consumption of arginine, small amounts of citrulline and ornithine were detected for both strains, of which higher concentrations were observed for 23K (S3 Table). These data reflect the activity of the arginine deiminase (ADI) pathway, in which arginine is converted via citrulline into ornithine, ammonia and CO_2_ with production of ATP [10, 11, 35–37]. About 98–99% of asparagine and glutamine were consumed by both strains at both growth rates, and 96% of serine (S2 Table).

### Proteome and transcriptome profiling

The effects of glucose availability on the proteome and transcriptome, hence growth rate-dependent relative protein and gene transcript levels, were investigated. The proteome was analyzed using high-resolution LTQ-Orbitrap mass spectrometry, where peptides of 756 and 906 cytoplasmic proteins were detected in all the 6 samples of 23K and LS25, respectively, and used for quantitation for each strain. Subsequent analysis of gene transcripts by RNA sequencing (RNA-seq) was performed, and for 23K and LS25 (excluding 23S and 16S genes), 99.9% and 99.7% of the genes were accounted for, respectively. The average number of reads per gene were 1124 and 642, median number of reads per gene were 179 and 172, and the average coverage per base inside genes were 84x and 47x for 23K and LS25, respectively. A complete set of proteome and transcriptome log_2_ transformed data can be found in S4 Table.

**Fig 3:**
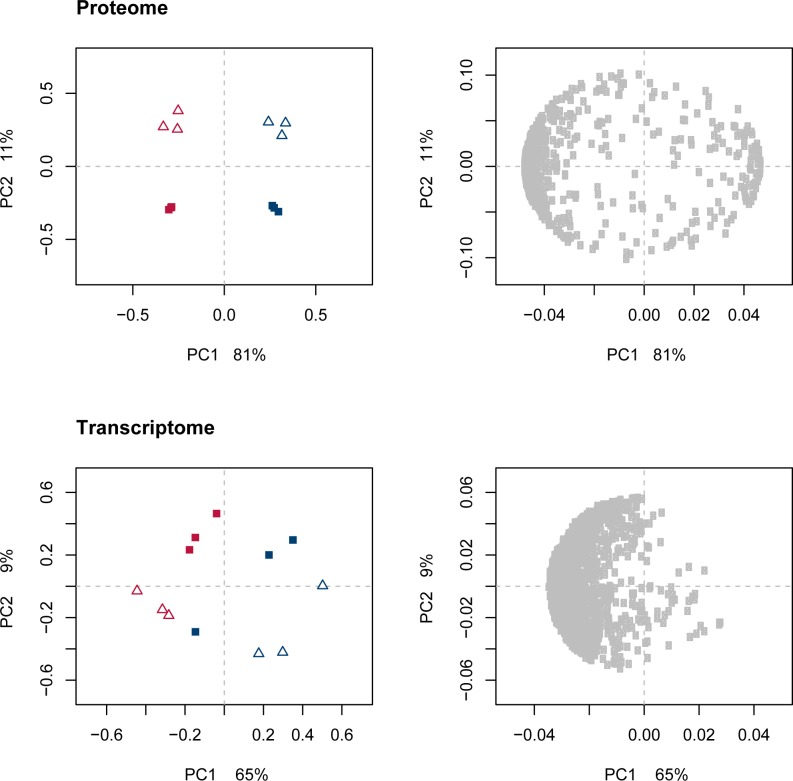
PCA on all features of the proteome and transcriptome. PCA of the proteome (all 643 variables) and transcriptome (all 1632 variables) mean centered and standardized to unit variance. Score plots (left) and loading plots (right) on PC1 (x-axis) vs PC2 (y-axis). *L*. *sakei* strains 23K and LS25 are shown in blue and red, respectively. Squares indicate high growth rate and high glucose availability. Open triangles indicate low growth rate and restricted glucose availability.

**Fig 4:**
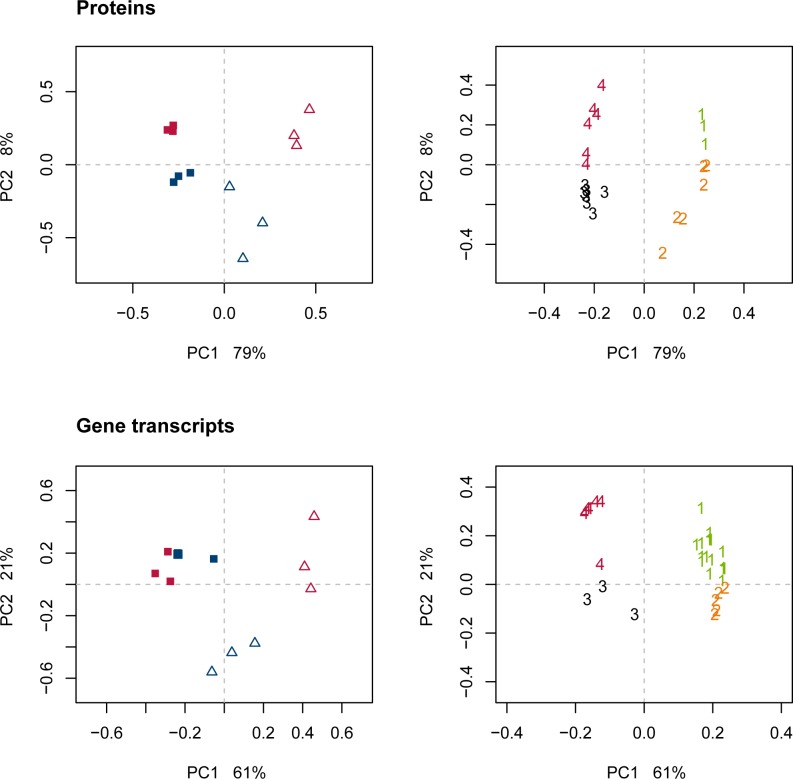
PCA of proteins and gene transcripts after variable selection towards the phenome. Proteins and gene transcripts selected by elastic net (left), where *L*. *sakei* strains 23K and LS25 are shown in blue and red, respectively. Squares indicate high growth rate and high glucose availability. Open triangles indicate low growth rate and restricted glucose availability. The selected proteins and transcripts were assigned to 4 groups with similar pattern (right), indicated in green, orange, black and red for groups 1–4, respectively.

By a multivariate approach, we analyzed how the proteome and transcriptome data relate to the changes in the phenome end-product profile. PCA on the proteome (Fig 3) distinguished well the effects of strain, PC1 explained by 81%, and the effects of growth rate, PC2 explained by 11%. PCA on the transcriptome (Fig 3) also defined the two strains as the most important variation in the data, with PC1 explained by 65%. The effect of growth rate shown in PC2 of the transcriptome was explained by 9%, and reflected differences between biological replicates as well. The expression of the majority of transcripts were higher for LS25 than for 23K (left side of the PCA loading plot, Fig 3). Further PC’s only implied differences within biological replicates (S1 and S2 Figs). Neither PCA of the proteome, nor of the transcriptome, reflected the same interaction pattern between strain and growth condition as seen for the phenome (Fig 2 and S1 Table). This suggested that the interaction pattern observed for the phenome with a stronger change for LS25 than for 23K was not associated with a corresponding variation in a large number of proteins nor transcripts. A minor group of proteins and transcripts were identified as relevant for the prediction of the phenome using supervised multivariate analysis by elastic net (S3 Fig), and univariate validation by confidence interval was used for omission of those that only described variation within biological replicates. PCA performed after this feature selection reflected similar pattern of variation as seen for the phenome, both for the proteins and for the transcripts (Fig 4, left), with a larger shift for LS25 than for 23K along the first PC. The selected features were assigned to 4 groups with similar pattern along the two first PCs (Fig 4, right). Plots of selected features log_2_ transformed data can be found in S4 Fig (proteome) and S5 Fig (transcriptome).

**Fig 5:**
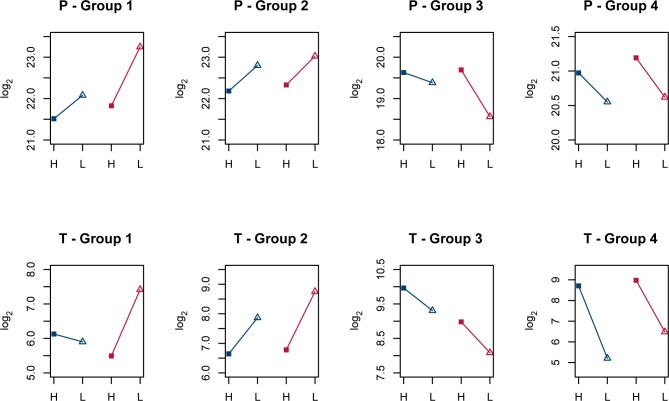
Mean expression of selected proteins and gene transcripts that responded to changed glucose availability in *L*. *sakei*. Expression of selected proteins (P) and gene transcripts (T) as mean values within each group (1–4) are according to Tables 2 and 3, respectively. Groups are defined by the PCs in the PCA shown in Fig 4. *L*. *sakei* strain 23K is shown in blue and strain LS25 in red. Squares indicate high growth rate and high glucose availability (H). Open triangles indicate low growth rate and restricted glucose availability (L).

Proteins with altered level of expression from high to low growth rate are presented in Table 2 and transcripts are presented in Table 3. Mean expression of the pattern of variation within each of the groups are illustrated in Fig 5.

**Table 2:** Proteins explaining *L*. *sakei* strain differences in phenome end-product profiles from high to low growth rate. Fold change from high to low growth rate and simple correlation coefficient (*r*) between selected protein and each end-product are shown. The proteins are assigned to 4 groups with similar pattern (Figs 4 and 5; S4 Fig).

	*L*. *sakei* locus tag			Fold change^a^		Correlation, *r*			
Group	23K	LS25	Protein	23K	LS25	lactate	formate	acetate	ethanol
**1**	LCA_0316	LS25_0363	L-serine dehydratase subunit beta (SDH)	0.38	**1.36**	-0.89	0.88	0.81	0.90
	LCA_0848	LS25_0936	Redox-sensing transcriptional repressor Rex	0.41	**1.23**	-0.93	0.93	0.89	0.96
	LCA_0974	LS25_1064	Formate C-acetyltransferase (PFL)	**0.91**	**1.67**	-0.90	0.90	0.84	0.93
**2**	LCA_0802	LS25_0894	NADH oxidase (Nox)	**0.74**	**1.09**	-0.95	0.94	0.93	0.95
	LCA_1154	LS25_1251	Hypothetical protein	**0.47**	**0.45**	-0.85	0.86	0.88	0.83
	LCA_1881	LS25_0004	Putative single-stranded nucleic acid binding protein	**0.64**	**0.54**	-0.88	0.88	0.90	0.88
**3**	LCA_0088	LS25_0092	Adenine deaminase	-0.59	**-2.11**	0.86	-0.86	-0.83	-0.87
	LCA_1099	LS25_1198	Folylpolyglutamate synthase	0.07	**-0.54**	0.60	-0.60	-0.53	-0.61
	LCA_1440	LS25_1514	CutC family copper homeostasis protein	-0.12	**-0.74**	0.87	-0.87	-0.82	-0.86
	LCA_1467	LS25_1541	Hypothetical protein	-0.53	**-2.09**	0.89	-0.88	-0.83	-0.89
	LCA_1559	LS25_1643	Putative oxidoreductase	-0.29	**-0.87**	0.87	-0.87	-0.84	-0.87
	LCA_1573	LS25_1658	Putative teichoic acid/polysaccharide glycosyl transferase, group 1	-0.15	**-0.54**	0.89	-0.89	-0.87	-0.85
	LCA_1879	LS25_0002	Cell division protein GidA	-0.11	**-1.01**	0.79	-0.78	-0.73	-0.75
**4**	LCA_0830	LS25_0920	Inosine-uridine preferring nucleoside hydrolase	**-0.71**	**-0.60**	0.75	-0.75	-0.81	-0.74
	LCA_0914	LS25_1000	Asparaginyl-tRNA synthetase	-0.05	**-0.41**	0.86	-0.86	-0.86	-0.83
	LCA_1373	LS25_1447	Uridine kinase	-0.37	**-0.45**	0.83	-0.84	-0.88	-0.83
	LCA_1421	LS25_1495	Arginyl-tRNA synthetase	**-0.45**	**-0.48**	0.82	-0.83	-0.88	-0.78
	LCA_1552	LS25_1635	ATP-dependent DNA helicase	**-0.54**	**-0.77**	0.58	-0.59	-0.70	-0.52
	LCA_1878	LS25_0001	Putative drug resistance ABC transporter, two ATP-binding subunits	-0.41	**-0.72**	0.69	-0.70	-0.77	-0.62

^a^Significant (p<0.05) fold change values are shown in bold writing

**Table 3:** Gene transcripts explaining *L*. *sakei* strain differences in phenome end-product profiles from high to low growth rate. Fold change from high to low growth rate and simple correlation coefficient (*r*) between selected transcript and each end-product are shown. The transcripts are assigned to 4 groups with similar pattern (Figs 4 and 5; S5 Fig).

	*L*. *sakei* locus tag				Fold change^a^		Correlation, *r*			
Group	23K	LS25		Gene product (*gene*)	23K	LS25	lactate	formate	acetate	ethanol
**1**	LCA_0195		LS25_0201	Hypothetical lipoprotein precursor	-0.49	**1.95**	-0.69	0.68	0.57	0.72
	LCA_0777		LS25_0869	Hypothetical protein	0.40	**1.78**	-0.79	0.78	0.76	0.75
	LCA_0802		LS25_0894	NADH oxidase (*nox*)	0.15	**0.84**	-0.73	0.72	0.64	0.73
	LCA_0831		LS25_0922	Putative nitroreductase, oxidoreductase	**-0.87**	**2.45**	-0.70	0.68	0.61	0.65
	LCA_0897		LS25_0973	Dipeptidase D-type, U34 family (*pepD5*)	-0.08	**2.17**	-0.78	0.77	0.74	0.73
	LCA_0930		LS25_1015	Putative ABC transporter, ATP-binding subunit	**-0.58**	**2.26**	-0.67	0.66	0.64	0.63
	LCA_1190		LS25_1281	Putative 4-carboxymuconolactone decarboxylase	-0.01	**1.12**	-0.77	0.76	0.70	0.78
	LCA_1191		LS25_1282	Hypothetical protein	0.32	**1.67**	-0.86	0.85	0.79	0.87
	LCA_1192		LS25_1283	Putative transcriptional regulator, MerR family	-0.17	**3.24**	-0.76	0.75	0.73	0.74
	LCA_1193		LS25_1284	Putative oxidoreductase	0.64	**2.15**	-0.84	0.83	0.79	0.84
	LCA_1287		LS25_1358	Hypothetical cell surface protein	**-0.95**	**1.62**	-0.59	0.57	0.46	0.59
	LCA_1512		LS25_1594	Putative polysaccharide biosynthesis protein, chain length determination	**-1.08**	**1.81**	-0.55	0.53	0.48	0.50
**2**	LCA_0509		LS25_0572	2-amino-3-ketobutyrate CoA ligase (*kbl*)	**2.34**	**2.34**	-0.83	0.84	0.81	0.85
	LCA_0510^b^		LS25_0573	L-threonine dehydrogenase	**1.81**	**2.43**	-0.88	0.89	0.87	0.89
	LCA_0742		LS25_0798	Transcriptional regulator MraZ	0.62	**1.73**	-0.73	0.72	0.74	0.68
	LCA_0743		LS25_0799	Putative S-adenosylmethionine-dependent-methyltransferase	0.75	**1.90**	-0.74	0.73	0.72	0.68
	LCA_1526		LS25_1609	Hypothetical protein	0.59	**1.47**	-0.83	0.82	0.79	0.79
**3**	LCA_0202		LS25_0208	Ribokinase (*rbsK*)	-0.65	**-0.89**	0.67	-0.67	-0.63	-0.68
**4**	LCA_0217		LS25_0262	Putative thiosulfate sulfurtransferase with a ArsR-HTH domain, Rhodanese family	**-2.53**	**-1.07**	0.54	-0.56	-0.66	-0.51
	LCA_0705		LS25_0753	Oligopeptide ABC transporter ATP-binding subunit (*oppD*)	**-4.10**	**-3.91**	0.70	-0.71	-0.79	-0.68
	LCA_0706		LS25_0754	Oligopeptide ABC transporter ATP-binding subunit (*oppF*)	**-4.31**	**-4.11**	0.69	-0.71	-0.79	-0.66
	LCA_0788		LS25_0881	Putative MIP family facilitator protein	**-3.81**	-1.91	0.61	-0.63	-0.70	-0.61
	LCA_0790		LS25_0883	Hypothetical protein	**-3.79**	-2.48	0.69	-0.71	-0.77	-0.68
	LCA_1787		LS25_1877	Hypothetical cell surface protein	**-2.47**	-1.50	0.69	-0.71	-0.76	-0.69

^a^Significant (p<0.05) fold change values are shown in bold writing

^b^N-terminal fragment, authentic frameshift

### Proteome responses to reduced glucose availability

Proteins in group 1 and group 2 (Fig 5, Table 2 and S4 Fig) displayed elevated levels at low compared with high growth rate, and particularly for group 1 proteins, the increase was stronger for LS25 than for 23K. These proteins were positively correlated to formate, acetate and ethanol, and negatively correlated to lactate, thus they increased as the fermentation was directed towards mixed acid production. One of the proteins in group 1, formate C-acetyltransferase, often called pyruvate formate lyase (PFL), increased for both strains. This enzyme catalyzes the reversible conversion of pyruvate and Coenzyme-A (CoA) into formate and acetyl-CoA. Among all the selected proteins, PFL increased the most. Another protein in this group, L-serine dehydratase (SDH) is an enzyme composed of two subunits (beta and alpha, respectively) which may deaminate the amino acid serine to yield pyruvate with the release of ammonia (NH_3_). The level of SDH beta subunit increased strongly for LS25. Redox-sensing transcriptional repressor Rex also increased strongly for LS25. This gene regulator is known to respond to an elevated NADH/NAD^+^ ratio by differential binding to Rex operators [38]. Among the proteins in group 2 was NADH oxidase (Nox), which can reoxidize excess NADH formed [4]. This enzyme displayed an increased expression for both strains, more for LS25 than for 23K. Elevated levels were also seen for single stranded nucleic acid binding protein and a hypothetical protein.

Proteins in group 3 and group 4 (Fig 5, Table 2 and S4 Fig) displayed a reduction at low compared with high growth rate for LS25, and for some of the group 4 proteins also for 23K. The proteins in group 3 comprised adenine deaminase involved in purine metabolism, a hypothetical protein, cell division protein GidA, putative oxidoreductase, CutC family copper homeostasis protein, putative teichoic acid/polysaccharide glycosyl transferase and folylpolyglutamate synthase. Among the proteins in group 4, inosine-uridine preferring nucleoside hydrolase, involved in nucleoside catabolism and responsible for the conversion of inosine to ribose and a purine base, was affected in both strains, as was arginyl-tRNA synthetase and ATP dependent DNA helicase as well. For LS25, reduced expression was also seen for uridine kinase involved in pyrimidine metabolism, asparginyl-tRNA synthetase and putative drug resistance ABC transporter.

### Transcriptome responses to reduced glucose availability

At low compared with high growth rate, gene transcripts in group 1 (Fig 5, Table 3 and S5 Fig) showed strongly elevated levels for LS25, and simultaneously, no change or lower levels were seen for 23K. Genes encoding putative nitroreductase, putative ABC transporter, hypothetical cell surface protein and putative polysaccharide biosynthesis protein showed this pattern of regulation. In addition, for LS25, four upregulated genes seemed to be subjected to coordinated regulation, encoding putative 4-carboxymuconolactone decarboxylase, hypothetical protein, putative MerR family transcriptional regulator and putative oxidoreductase. The *nox* gene was also upregulated for LS25, and so were genes encoding hypothetical lipoprotein precursor, hypothetical protein and dipeptidase U34. Transcripts in group 2 (Fig 5, Table 3 and S5 Fig) showed increased levels. Increases for both strains were seen for *kbl* encoding the enzyme 2-amino-3-ketobutyrate CoA ligase, also named glycine C-acetyltransferase, which catalyzes the chemical reaction of acetyl-CoA and glycine into CoA and 2-amino-3-oxobutanoate and vice versa. Likely subjected to coordinated regulation with *kbl*, both strains also upregulated the gene encoding L-threonine dehydrogenase, that facilitates the catabolism of threonine by catalyzing conversion to glycine via 2-amino-3-ketobutyrate with concomitant reduction of NAD^+^. The gene is annotated with a nonsense mutation in 23K [1], confirmed by examining the RNA-seq reads (not shown), while it is complete in LS25 [19]. Moreover, for LS25, three more upregulated genes in group 2 encode hypothetical protein, transcriptional regulator MraZ and putative S-adenosylmethionine-dependent-methyltransferase, and the two latter seemed to be subjected to coordinated regulation.

Gene transcripts in group 3 and group 4 (Fig 5, Table 3 and S5 Fig) displayed lower levels at low compared with high growth rate. The group 3 gene *rbsK* encodes the ribokinase involved in ribose catabolism. Among genes in group 4, *oppD* and *oppF* were strongly downregulated for both strains. Opp is an ABC transporter encoded by the *oppABDCDF* operon, and is composed of substrate-binding lipoprotein OppA that binds extracellular peptides, two membrane-spanning transporter permeases, OppB and OppC, and two cytoplasmic ATPases, OppD and OppF. The gene encoding putative thiosulfate sulfurtransferase was also downregulated for both strains, whereas several transcripts displayed lower level only for 23K, including putative MIP family facilitator protein, hypothetical protein and hypothetical cell surface protein.

## Discussion

We have investigated the effect of glucose availability in *L*. *sakei* strains 23K and LS25 on the metabolite, proteome and transcriptome levels, where different degrees of response to the same energy restriction revealed strain specific regulation. Both strains shifted from homolactic fermentation at high growth rate towards more mixed acid fermentation at low growth rate, also observed by others [13, 16, 24, 39] and a common phenome in LAB [4]. However, the change was more pronounced for LS25 than for 23K with reduced glucose availability.

The observed metabolic change is attributed to an altered pyruvate metabolism which benefit the bacteria by generating ATP, or by gaining NAD^+^ for maintaining the redox balance, where various end-products in addition to lactate are produced [4]. Elevated production level of formate, more pronounced for LS25, was clearly reflected in the increased expression of PFL. Only active anaerobically, the PFL system [40, 41] was shown induced with lowering of the dilution rate in continuous culture systems also by others [26]. Between various organisms, shifts in metabolic strategies during growth are regulated in different ways, among which intracellular redox potential reflected by NADH/NAD^+^ ratio have been reported for LAB to be a key sensor [13, 42]. Various regulatory sensors monitor the redox state of all the cellular components for optimal overall function [43]. The transcriptional regulator Rex responds to NADH/NAD^+^ levels and negatively controls expression of genes involved in energy metabolism and fermentative growth in Gram-positive bacteria [38, 44–47]. Elevated protein level, stronger for LS25 than for 23K during slow growth, suggests this regulatory role for Rex. The commonality among the Rex-regulated enzymes is that they all function to maintain the NADH/NAD^+^ ratio needed to reposition the cells for re-entry into glycolysis. In response to carbohydrate limitations and/or high intracellular NADH/NAD^+^ level, the bacterial cells divert the catabolic pathways away from homofermentation towards mixed acid fermentation. Under low NADH/NAD^+^ ratio, Rex protein binds to the target sites and represses transcription of genes involved in NADH reoxidation, while the increase of NADH concentration results in the dissociation of Rex from DNA and thus derepression of its target genes [46, 48]. By comparative genomics approach, Ravcheev *et al*. [48] inferred 6 candidate Rex-binding sites located upstream of *L*. *sakei* target genes and operons. This included the Rex encoding gene, and among our selected features, the *nox* gene. Upregulated *nox* observed for LS25 during slow growth, as well as elevated Nox levels for both strains, but more pronounced for LS25, could imply regulation by Rex.

The observation that as much as 99% of the variation in the phenome could be accounted for by only one linear latent variable on the phenome level, with a stronger effect for LS25 than for 23K, suggests that a simple underlying mechanism could be controlling the effect. Potentially, there may be only one primary mechanism that secondary lead to coordinated changes. Rex is a candidate for such a primary effect. The elevated level of Nox is dually beneficial with alternative routes for NAD^+^ regeneration, and for detoxifying deleterious oxygen metabolites [1]. Despite a predilection for anaerobiosis, *L*. *sakei* is surprisingly well equipped to cope with changing oxygen and redox levels [1]. Several genes encoding proteins with oxidoreductase activity upregulated for LS25 also reflect the need to maintain the redox balance. A large arsenal of putative oxidoreductases with few homologs among lactobacilli are identified in *L*. *sakei*, indicating an efficient capacity for maintaining redox balance through tight control of the NADH/NAD^+^ ratio and the use of various electron acceptors [1].

Interestingly, our present experiment could be considered as an analog for *L*. *sakei* to the extensively studied topic of so-called complete calorie restricted diet, although in the present study glucose was the limiting factor. Complete caloric restriction is shown as a conserved mechanism in eukaryotes, from single-celled yeast to humans, to result in expanded healthy life span in response to a reduction of energy intake [49–52], and is found to act through mechanisms involving the redox balance [53]. For *L*. *sakei*, serine and threonine possibly enter the pyruvate pool after enzymatic conversion by SDH and L-threonine dehydrogenase at restricted glucose availability. Indeed, the level of SDH increased at low growth rate in LS25, and higher consumption of threonine was detected. The L-threonine dehydrogenase gene transcripts were upregulated in both strains at low growth rate. This gene contains a nonsense mutation in 23K [1]. However, the gene product(s) may still form an active enzyme. Complete caloric restricted diets in eukaryotes leads to elevated levels of gluconeogenic enzymes and transaminases of several amino acids [54], and specifically it has been shown that SDH is critical for serine utilization in gluconeogenesis [55].

An interesting difference between the two strains seemed to lay in the catabolism of arginine. Consumption of this amino acid and concominant production of citrulline and ornithine reflected the activity of the ADI pathway, seemingly more active in 23K than in LS25. The ability to utilize arginine as an energy source to generate ATP, which represents a competitive benefit in the low carbohydrate containing meat environment, has been thoroughly studied in *L*. *sakei*, however only for a few strains [10, 11, 35–37, 56, 57]. A second putative ADI pathway (LCA_0067–0073), present in 23K and suggested to further enhance its ability to survive in the meat environment [1], is not present in LS25 [19, 23]. By comparative genome hybridization using a microarray based on the 23K genome, this second putative ADI pathway was reported present in 10 of 18 *L*. *sakei* strains investigated [23]. Hence this second pathway may play a role in arginine catabolism under certain conditions. Mixed acid fermentation yields one extra ATP per glucose over homolactic fermentation [4]. Concominant with this higher ATP yield at slow growth, the bacterium also seemed to downregulate various pathways for saving energy, as seen for both strains in lower level of the ATP dependent helicase and proteins involved in purine/pyrimidine metabolism, as well as downregulation of genes encoding membrane proteins involved in oligopeptide uptake (*opp* genes) and glycerol uptake (MIP family facilitator protein). Biomass yield in *L*. *sakei* declined in slow growing cells. This might appear counterintuitive at first, since at slower growth rates cells adopt a metabolic strategy that results in a higher ATP yield per glucose consumed. However, this might not necessarily manifest itself as higher biomass yield since the gain in efficiency on the catabolism of the substrate, can be (and often is) offset by the increasing proportion of the cellular ATP requirements that get allocated from growth to maintenance (e.g. protein and nucleic acid repair) [58, 59].

It is clear from both the proteome and transcriptome changes in response to glucose availability that maintaining the redox balance is crucial for the optimum growth and survival of *L*. *sakei*. However, strain variation exists on how the redox and energy state are sensed and regulated to ensure an optimal outcome for the different cells. Investigations of a larger set of strains could elucidate whether the variation is strain-specific or specific to the two different subspecies, *carnosus* and *sakei*.

## Supporting information

S1 Table Effects of strain, growth condition and interaction effects (strain*growth) for end-products and amino acids. *L*. *sakei* strains 23K and LS25 were grown in glucose-limited CDM-LAB medium at high and low growth rates. FDR-adjusted p-values are shown. Asterix (*) indicates significant change (p.FDR<0.05).
(PDF)

S2 Table Amino acid consumption in *L*. *sakei* during continuous cultivation in glucose-limited CDM-LAB medium at different growth rates. The consumption is shown in mM and %.
(PDF)

S3 Table Production of citrulline and ornithine in *L*. *sakei* during continuous cultivation in glucose-limited CDM-LAB medium at different growth rates. (PDF)

S4 Table Proteome and transcriptome log_2_ transformed data. *L*. *sakei* common genes, unique genes for strain 23K and unique genes for strain LS25 are listed by locus tags (LCA_ for strain 23K and LS25_ for strain LS25). Each sample set included 3 samples at high (H) and 3 samples at low (L) growth rate with 3 technical replicates (a, b, c) for the proteome (P) and transcriptome (T) analyses.
(XLSX)

S1 Fig PCA on all features of the proteome. PCA of the proteome (all 643 variables) mean centered and standardized to unit variance. Score plots (left) and loading plots (right) on PC3 (x-axis) vs PC4 (y-axis) and PC5 (x-axis) vs PC6 (y-axis). *L*. *sakei* strains 23K and LS25 are shown in blue and red, respectively. Squares indicate high growth rate and open triangles indicate low growth rate.
(PDF)

S2 Fig PCA on all features of the transcriptome. PCA of the transcriptome (all 1632 variables) mean centered and standardized to unit variance. Score plots (left) and loading plots (right) on PC3 (x-axis) vs PC4 (y-axis) and PC5 (x-axis) vs PC6 (y-axis). *L*. *sakei* strains 23K and LS25 are shown in blue and red, respectively. Squares indicate high growth rate and open triangles indicate low growth rate.
(PDF)

S3 Fig Elastic net coefficient and cross-validation curves. Coefficient curves (left plots) display the coefficients of each variable (the proteome or the transcriptome) in different colors with increasing number of variables in the model as the regularization parameter and thereby the L1-norm (sum of the absolute value of regression coefficients) changes. Cross-validation curves (right plots) are displayed in red, upper and lower standard deviation curves in grey, for the prediction of the phenome (lactate, formate, acetate and ethanol) based on input variables. MSE refers to Mean Square Error.
(PDF)

S4 Fig Proteins that responded to changed glucose availability in *L*. *sakei*. The proteins (P) are listed by 23K locus tag (LCA_XXXX), protein name and group according to Table 2. Strains 23K and LS25 are shown in blue and red, respectively. Squares indicate high growth rate and open triangles indicate low growth rate. The proteins were selected by elastic net repeated 1000 times using alpha tuning parameter 0.5 and regularization parameter lambda set to log.lambda.min, followed by confidence intervals within each strain.
(PDF)

S5 Fig Gene transcript that responded to changed glucose availability in *L*. *sakei*. The gene transcripts (T) are listed by 23K locus tag (LCA_XXXX), gene product name and group according to Table 3. Strains 23K and LS25 are shown in blue and red, respectively. Squares indicate high growth rate and high glucose availability. Open triangles indicate low growth rate and restricted glucose availability. The gene transcripts were selected by elastic net repeated 1000 times using alpha tuning parameter 0.5 and regularization parameter lambda set to log.lambda.min, followed by confidence intervals within each strain.
(PDF)

## References

[1] Chaillou S, Champomier-Verges MC, Cornet M, Crutz-Le Coq AM, Dudez AM, Martin V, et al The complete genome sequence of the meat-borne lactic acid bacterium *Lactobacillus sakei* 23K. Nat Biotechnol. 2005; 23(12):1527–1533. doi: 10.1038/nbt1160 16273110 10.1038/nbt1160

[2] Champomier-Verges MC, Chaillou S, Cornet M, Zagorec M. Erratum to "*Lactobacillus sakei*: recent developments and future prospects". Res Microbiol. 2002; 153(2):115–123. 11900264 10.1016/s0923-2508(01)01296-7

[3] Lauret R, Morel-Deville F, Berthier F, Champomier-Verges M, Postma P, Ehrlich SD, et al Carbohydrate utilization in *Lactobacillus sake*. Appl Environ Microbiol. 1996; 62(6):1922–1927. 16535331 10.1128/aem.62.6.1922-1927.1996 PMC1388869

[4] Axelsson L. Lactic acid bacteria: classification and physiology In: Salminen S, von Wright A, Ouwehand A, editors. Lactic acid bacteria: microbiological and functional aspects. Third revised and expanded ed. New York, USA: Marcel Dekker, Inc./CRC Press; 2004 p. 1–66.

[5] Fadda S, Sanz Y, Vignolo G, Aristoy M, Oliver G, Toldra F. Hydrolysis of pork muscle sarcoplasmic proteins by *Lactobacillus curvatus* and *Lactobacillus sake*. Appl Environ Microbiol. 1999; 65(2):578–584. 9925585 10.1128/aem.65.2.578-584.1999 PMC91064

[6] Sanz Y, Fadda S, Vignolo G, Aristoy MC, Oliver G, Toldra F. Hydrolysis of muscle myofibrillar proteins by *Lactobacillus curvatus* and *Lactobacillus sake*. Int J Food Microbiol. 1999; 53(2–3):115–125. 10634703 10.1016/s0168-1605(99)00134-8

[7] McLeod A, Snipen L, Naterstad K, Axelsson L. Global transcriptome response in *Lactobacillus sakei* during growth on ribose. BMC Microbiol. 2011; 11:145 doi: 10.1186/1471-2180-11-145 21702908 10.1186/1471-2180-11-145 PMC3146418

[8] McLeod A, Zagorec M, Champomier-Verges MC, Naterstad K, Axelsson L. Primary metabolism in *Lactobacillus sakei* food isolates by proteomic analysis. BMC Microbiol. 2010; 10:120 doi: 10.1186/1471-2180-10-120 20412581 10.1186/1471-2180-10-120 PMC2873491

[9] Rimaux T, Vrancken G, Vuylsteke B, De Vuyst L, Leroy F. The pentose moiety of adenosine and inosine is an important energy source for the fermented-meat starter culture *Lactobacillus sakei* CTC 494. Appl Environ Microbiol. 2011; 77(18):6539–6550. doi: 10.1128/AEM.00498-11 21803903 10.1128/AEM.00498-11 PMC3187176

[10] Champomier Verges MC, Zuniga M, Morel-Deville F, Perez-Martinez G, Zagorec M, Ehrlich SD. Relationships between arginine degradation, pH and survival in *Lactobacillus sakei*. FEMS Microbiol Lett. 1999; 180(2):297–304. 10556725 10.1111/j.1574-6968.1999.tb08809.x

[11] Montel MC, Champomier MC. Arginine catabolism in *Lactobacillus sake* isolated from meat. Appl Environ Microbiol. 1987; 53(11):2683–2685. 3426226 10.1128/aem.53.11.2683-2685.1987 PMC204175

[12] Novick A, Szilard L. Description of the chemostat. Science. 1950; 112(2920):715–716. 14787503 10.1126/science.112.2920.715

[13] Goel A, Wortel MT, Molenaar D, Teusink B. Metabolic shifts: a fitness perspective for microbial cell factories. Biotechnol Lett. 2012; 34(12):2147–2160. doi: 10.1007/s10529-012-1038-9 22936303 10.1007/s10529-012-1038-9 PMC3487007

[14] Huberts DH, Niebel B, Heinemann M. A flux-sensing mechanism could regulate the switch between respiration and fermentation. FEMS Yeast Res. 2012; 12(2):118–128. doi: 10.1111/j.1567-1364.2011.00767.x 22129078 10.1111/j.1567-1364.2011.00767.x

[15] Sauer U, Eikmanns BJ. The PEP-pyruvate-oxaloacetate node as the switch point for carbon flux distribution in bacteria. FEMS Microbiol Rev. 2005; 29(4):765–794. doi: 10.1016/j.femsre.2004.11.002 16102602 10.1016/j.femsre.2004.11.002

[16] Thomas TD, Ellwood DC, Longyear VM. Change from homo- to heterolactic fermentation by *Streptococcus lactis* resulting from glucose limitation in anaerobic chemostat cultures. J bacteriol. 1979; 138(1):109–17. 108249 10.1128/jb.138.1.109-117.1979 PMC218245

[17] Berthier F, Zagorec M, Champomier-Verges M, Ehrlich SD, Morel-Deville F. Efficient transformation of *Lactobacillus sake* by electroporation. Microbiol-Uk. 1996; 142:1273–1279. 10.1099/13500872-142-5-1273 33725790

[18] Hagen BF, Naes H, Holck AL. Meat starters have individual requirements for Mn(2+). Meat Sci. 2000;55(2):161–8. 22061081 10.1016/s0309-1740(99)00138-2

[19] McLeod A, Brede DA, Rud I, Axelsson L. Genome sequence of *Lactobacillus sakei* subsp. *sakei* LS25, a commercial starter culture strain for fermented sausage. Genome Announc. 2013; 1(4). 10.1128/genomeA.00475-13 PMC3709151 23846274

[20] Klein G, Dicks LMT, Pack A, Hack B, Zimmerman K, Dellaglio F, et al Emended description of *Lactobacillus sake* (Katahiri, Katahara and Fukami) and *Lactobacillus curvatus* (Abo-Elnega Kandler): Numerical classification revealed by protein fingerprinting and identification based on biochemical patterns and DNA–DNA hybridizations. Int J Syst Bacteriol. 1996; 46:367–376.

[21] McLeod A, Nyquist OL, Snipen L, Naterstad K, Axelsson L. Diversity of *Lactobacillus sakei* strains investigated by phenotypic and genotypic methods. Syst Appl Microbiol. 2008; 31(5):393–403. doi: 10.1016/j.syapm.2008.06.002 18678454 10.1016/j.syapm.2008.06.002

[22] Torriani S, Van Reenen GA, Klein G, Reuter G, Dellaglio F, Dicks LM. *Lactobacillus curvatus* subsp. *curvatus* subsp. nov. and *Lactobacillus curvatus* subsp. *melibiosus* subsp. nov. and *Lactobacillus sake* subsp. *sake* subsp. nov. and *Lactobacillus sake* subsp. *carnosus* subsp. nov., new subspecies of *Lactobacillus curvatus* Abo-Elnaga and Kandler 1965 and *Lactobacillus sake* Katagiri, Kitahara, and Fukami 1934 (Klein et al. 1996, emended descriptions), respectively. Int J Syst Bacteriol. 1996; 46(4):1158–1163. doi: 10.1099/00207713-46-4-1158 8863451 10.1099/00207713-46-4-1158

[23] Nyquist OL, McLeod A, Brede DA, Snipen L, Aakra A, Nes IF. Comparative genomics of *Lactobacillus sakei* with emphasis on strains from meat. MGG Mol Genet Genomics. 2011; 285(4):297–311. doi: 10.1007/s00438-011-0608-1 21369871 10.1007/s00438-011-0608-1

[24] Fiedler T, Bekker M, Jonsson M, Mehmeti I, Pritzschke A, Siemens N, et al Characterization of three lactic acid bacteria and their isogenic *ldh* deletion mutants shows optimization for YATP (cell mass produced per mole of ATP) at their physiological pHs. Appl Environ Microbiol. 2011; 77(2):612–7. doi: 10.1128/AEM.01838-10 21097579 10.1128/AEM.01838-10 PMC3020547

[25] Jonsson M, Saleihan Z, Nes IF, Holo H. Construction and characterization of three lactate dehydrogenase-negative *Enterococcus faecalis* V583 mutants. Appl Environ Microbiol. 2009; 75(14):4901–3. doi: 10.1128/AEM.00344-09 19465534 10.1128/AEM.00344-09 PMC2708445

[26] Goel A, Eckhardt TH, Puri P, de Jong A, Santos FB, Giera M, et al Protein costs do not explain evolution of metabolic strategies and regulation of ribosomal content. Mol Microbiol. 2015; 97(1):77–92. doi: 10.1111/mmi.13012 25828364 10.1111/mmi.13012

[27] Vaudel M, Barsnes H, Berven FS, Sickmann A, Martens L. SearchGUI: An open-source graphical user interface for simultaneous OMSSA and X!Tandem searches. Proteomics. 2011; 11(5):996–999. doi: 10.1002/pmic.201000595 21337703 10.1002/pmic.201000595

[28] Rud I, Naterstad K, Bongers RS, Molenaar D, Kleerebezem M, Axelsson L. Functional analysis of the role of CggR (central glycolytic gene regulator) in *Lactobacillus plantarum* by transcriptome analysis. Microb Biotechnol. 2011; 4(3):345–356. doi: 10.1111/j.1751-7915.2010.00223.x 21375718 10.1111/j.1751-7915.2010.00223.x PMC3818993

[29] Langmead B, Trapnell C, Pop M, Salzberg SL. Ultrafast and memory-efficient alignment of short DNA sequences to the human genome. Genome Biol. 2009; 10(3):R25 doi: 10.1186/gb-2009-10-3-r25 19261174 10.1186/gb-2009-10-3-r25 PMC2690996

[30] Langsrud Ø. 50–50 multivariate analysis of variance for collinear responses. The Statistician. 2002;51:305–317.

[31] Zou H, Hastie T. Regularization and variable selection via the elastic net. J Roy Stat Soc B. 2005; 67:301–320.

[32] Breiman L. Better subset regression using the nonnegative garrote. Technometrics. 1995; 37(4):373–384.

[33] Tibshirani R. Regression shrinkage and selection via the Lasso. J Roy Stat Soc B Met. 1996; 58(1):267–288.

[34] Neyman J. Outline of a theory of statistical estimation based on the classical theory of probability. Philos Trans R Soc A. 1937; 236:333–380.

[35] Rimaux T, Vrancken G, Pothakos V, Maes D, De Vuyst L, Leroy F. The kinetics of the arginine deiminase pathway in the meat starter culture *Lactobacillus sakei* CTC 494 are pH-dependent. Food Microbiol. 2011; 28(3):597–604. doi: 10.1016/j.fm.2010.11.016 21356470 10.1016/j.fm.2010.11.016

[36] Zuniga M, Champomier-Verges M, Zagorec M, Perez-Martinez G. Structural and functional analysis of the gene cluster encoding the enzymes of the arginine deiminase pathway of *Lactobacillus sake*. J Bacteriol. 1998; 180(16):4154–4159. 9696763 10.1128/jb.180.16.4154-4159.1998 PMC107411

[37] Zuniga M, Miralles Md Mdel C, Perez-Martinez G. The Product of *arcR*, the sixth gene of the *arc* operon of *Lactobacillus sakei*, is essential for expression of the arginine deiminase pathway. Appl Environ Microbiol. 2002; 68(12):6051–6058. doi: 10.1128/AEM.68.12.6051-6058.2002 12450828 10.1128/AEM.68.12.6051-6058.2002 PMC134381

[38] Fuchs S, Pane-Farre J, Kohler C, Hecker M, Engelmann S. Anaerobic gene expression in *Staphylococcus aureus*. J Bacteriol. 2007; 189(11):4275–4289. doi: 10.1128/JB.00081-07 17384184 10.1128/JB.00081-07 PMC1913399

[39] Mehmeti I, Faergestad EM, Bekker M, Snipen L, Nes IF, Holo H. Growth rate-dependent control in *Enterococcus faecalis*: effects on the transcriptome and proteome, and strong regulation of lactate dehydrogenase. Appl Environ Microbiol. 2012; 78(1):170–176. doi: 10.1128/AEM.06604-11 22038603 10.1128/AEM.06604-11 PMC3255604

[40] Takahashi S, Abbe K, Yamada T. Purification of pyruvate formate-lyase from *Streptococcus mutans* and its regulatory properties. J Bacteriol. 1982; 149(3):1034–1040. 7061379 10.1128/jb.149.3.1034-1040.1982 PMC216493

[41] Thomas TD, Turner KW. Carbohydrate fermentation by *Streptococcus cremoris* and *Streptococcus lactis* growing in agar gels. Appl Environ Microbiol. 1981; 41(6):1289–1294. 16345783 10.1128/aem.41.6.1289-1294.1981 PMC243913

[42] van Hoek MJ, Merks RM. Redox balance is key to explaining full vs. partial switching to low-yield metabolism. BMC Syst Biol. 2012; 6:22 doi: 10.1186/1752-0509-6-22 22443685 10.1186/1752-0509-6-22 PMC3384451

[43] Green J, Paget MS. Bacterial redox sensors. Nature Rev Microbiol. 2004; 2(12):954–966. 15550941 10.1038/nrmicro1022

[44] Bitoun JP, Nguyen AH, Fan Y, Burne RA, Wen ZT. Transcriptional repressor Rex is involved in regulation of oxidative stress response and biofilm formation by *Streptococcus mutans*. FEMS Microbiol Lett. 2011; 320(2):110–117. doi: 10.1111/j.1574-6968.2011.02293.x 21521360 10.1111/j.1574-6968.2011.02293.x PMC3115380

[45] Mehmeti I, Jonsson M, Fergestad EM, Mathiesen G, Nes IF, Holo H. Transcriptome, proteome, and metabolite analyses of a lactate dehydrogenase-negative mutant of *Enterococcus faecalis* V583. Appl Environ Microbiol. 2011; 77(7):2406–2413. doi: 10.1128/AEM.02485-10 21296946 10.1128/AEM.02485-10 PMC3067458

[46] Pagels M, Fuchs S, Pane-Farre J, Kohler C, Menschner L, Hecker M, et al Redox sensing by a Rex-family repressor is involved in the regulation of anaerobic gene expression in *Staphylococcus aureus*. Mol Microbiol. 2010; 76(5):1142–1161. doi: 10.1111/j.1365-2958.2010.07105.x 20374494 10.1111/j.1365-2958.2010.07105.x PMC2883068

[47] Wang E, Bauer MC, Rogstam A, Linse S, Logan DT, von Wachenfeldt C. Structure and functional properties of the *Bacillus subtilis* transcriptional repressor Rex. Mol Microbiol. 2008; 69(2):466–478. doi: 10.1111/j.1365-2958.2008.06295.x 18485070 10.1111/j.1365-2958.2008.06295.x

[48] Ravcheev DA, Li X, Latif H, Zengler K, Leyn SA, Korostelev YD, et al Transcriptional regulation of central carbon and energy metabolism in bacteria by redox-responsive repressor Rex. J Bacteriol. 2012; 194(5):1145–1157. doi: 10.1128/JB.06412-11 22210771 10.1128/JB.06412-11 PMC3294762

[49] Barger JL, Anderson RM, Newton MA, da Silva C, Vann JA, Pugh TD, et al A conserved transcriptional signature of delayed aging and reduced disease vulnerability is partially mediated by SIRT3. PLoS ONE. 2015; 10(4):e0120738 doi: 10.1371/journal.pone.0120738 25830335 10.1371/journal.pone.0120738 PMC4382298

[50] Fontana L, Partridge L. Promoting health and longevity through diet: from model organisms to humans. Cell. 2015; 161(1):106–118. doi: 10.1016/j.cell.2015.02.020 25815989 10.1016/j.cell.2015.02.020 PMC4547605

[51] Ruetenik A, Barrientos A. Dietary restriction, mitochondrial function and aging: from yeast to humans. Bba-Bioenergetics. 2015; 1847(11):1434–1447. doi: 10.1016/j.bbabio.2015.05.005 25979234 10.1016/j.bbabio.2015.05.005 PMC4575837

[52] Weindruch R, Naylor PH, Goldstein AL, Walford RL. Influences of aging and dietary restriction on serum thymosin alpha 1 levels in mice. J Gerontol. 1988; 43:40–42. 10.1093/geronj/43.2.b40 3346517

[53] Luo H, Chiang HH, Louw M, Susanto A, Chen D. Nutrient sensing and the oxidative stress response. Trends Endocrinol Metab. 2017; 28(6):449–460. doi: 10.1016/j.tem.2017.02.008 28314502 10.1016/j.tem.2017.02.008 PMC5438757

[54] Hagopian K, Ramsey JJ, Weindruch R. Caloric restriction increases gluconeogenic and transaminase enzyme activities in mouse liver. Exp Gerontol. 2003; 38(3):267–278. 12581790 10.1016/s0531-5565(02)00202-4

[55] Hagopian K, Ramsey JJ, Weindruch R. Serine utilization in mouse liver: Influence of caloric restriction and aging. Febs Lett. 2005; 579(9):2009–2013. doi: 10.1016/j.febslet.2005.02.062 15792811 10.1016/j.febslet.2005.02.062

[56] Rimaux T, Riviere A, Hebert EM, Mozzi F, Weckx S, De Vuyst L, et al A putative transport protein is involved in citrulline excretion and re-uptake during arginine deiminase pathway activity by *Lactobacillus sakei*. Res Microbiol. 2013; 164(3):216–225. doi: 10.1016/j.resmic.2012.11.004 23178175 10.1016/j.resmic.2012.11.004

[57] Rimaux T, Riviere A, Illeghems K, Weckx S, De Vuyst L, Leroy F. Expression of the arginine deiminase pathway genes in *Lactobacillus sakei* is strain dependent and is affected by the environmental pH. Appl Environ Microbiol. 2012; 78(14):4874–4883. doi: 10.1128/AEM.07724-11 22544250 10.1128/AEM.07724-11 PMC3416364

[58] Bachmann H, Molenaar D, Branco Dos Santos F, Teusink B: Experimental evolution and the adjustment of metabolic strategies in lactic acid bacteria. FEMS Microbiol Rev. 2017; 41(Supp_1):201–219. 10.1093/femsre/fux024 28633473

[59] Lipson DA: The complex relationship between microbial growth rate and yield and its implications for ecosystem processes. Front Microbiol. 2015; 6:615 doi: 10.3389/fmicb.2015.00615 26136742 10.3389/fmicb.2015.00615 PMC4468913

