# Supplementary material for: Effects of glucose availability in Lactobacillus sakei; metabolic change and regulation of the proteome and transcriptome
Source: PLoS One. 2017 Nov 3;12(11):e0187542. doi: 10.1371/journal.pone.0187542 (PMC5669474; doi:10.1371/journal.pone.0187542)
Supplement: S1 Table — L. sakei strains 23K and LS25 were grown in glucose-limited CDM-LAB medium at high and low growth rates. FDR-adjusted p-values are shown. Asterix (*) indicates significant change (p.FDR<0.05). (PDF) [file pone.0187542.s001.pdf]

S1 Table. Effects of strain, growth condition and interaction effects (strain*growth) for end-products and amino acids. *L. sakei* strains 23K and LS25 were grown in glucose-limited CDM-LAB medium at high and low growth rates. FDR-adjusted p-values are shown. Asterix (*) indicates significant change (p.FDR<0.05).

		FDR adjusted p-values		
		**Strain**	**Growth**	**Strain*Growth**
**End-product**	**Lactate**	0.001*	0.000*	0.000*
	**Formate**	0.001*	0.000*	0.000*
	**Acetate**	0.123	0.000*	0.003*
	**Ethanol**	0.001*	0.000*	0.000*
**Amino acid**	**Asparagine**	0.332	0.159	0.347
	**Aspartic acid**	0.332	0.557	0.560
	**Arginine**	0.000*	0.334	0.581
	**Alanine**	0.534	0.003*	0.708
	**Glutamic acid**	0.360	0.717	0.580
	**Glutamine**	0.176	0.003*	0.140
	**Glycine**	0.831	0.263	0.629
	**Histidine**	0.537	0.116	0.767
	**Isoleucine**	0.332	0.255	0.525
	**Leucine**	0.659	0.096	0.851
	**Lysine**	0.534	0.048*	0.779
	**Methionine**	0.760	0.099	0.851
	**Phenylalanine**	0.401	0.147	0.295
	**Serine**	0.332	0.772	0.071
	**Threonine**	0.008*	0.003*	0.024*
	**Tryptophan**	0.663	0.048*	0.767
	**Tyrosine**	0.407	0.255	0.152
	**Valine**	0.537	0.380	0.525
