# Supplementary material for: Effects of glucose availability in Lactobacillus sakei; metabolic change and regulation of the proteome and transcriptome
Source: PLoS One. 2017 Nov 3;12(11):e0187542. doi: 10.1371/journal.pone.0187542 (PMC5669474; doi:10.1371/journal.pone.0187542)
Supplement: S2 Table — The consumption is shown in mM and %. (PDF) [file pone.0187542.s002.pdf]

S2 Table. Amino acid consumption in *L. sakei* during continuous cultivation in glucose-limited CDM-LAB medium at different growth rates. The consumption is shown in mM and %.

	Strain 23K				Strain LS25			
	mM consumed[a]		% consumed[a]		mM consumed[a]		% consumed[a]	
	0.357 h^{-1}	0.045 h^{-1}	0.357 h^{-1}	0.045 h^{-1}	0.357 h^{-1}	0.045 h^{-1}	0.357 h^{-1}	0.045 h^{-1}
**Asparagine**	0.61 (0.00)	0.60 (0.00)	99.27 (0.05)	98.90 (0.54)	0.61 (0.00)	0.61 (0.00)	99.28 (0.05)	99.25 (0.03)
**Aspartic acid**	0.96 (0.13)	1.05 (0.10)	40.02 (5.54)	43.86 (4.30)	0.87 (0.27)	0.64 (0.23)	36.39 (11.27)	26.71 (9.62)
**Arginine**	0.57 (0.00)	0.58 (0.00)	93.27 (0.69)	93.81 (0.62)	0.29 (0.04)	0.32 (0.00)	46.51 (6.22)	51.67 (0.32)
**Alanine**	0.98 (0.04)	0.63 (0.06)	39.61 (1.47)	25.32 (2.41)	0.91 (0.04)	0.72 (0.17)	36.74 (1.42)	29.02 (6.98)
**Glutamic acid**	0.74 (0.12)	0.88 (0.17)	22.18 (3.69)	26.27 (5.03)	0.66 (0.28)	0.50 (0.16)	19.93 (8.38)	14.95 (4.77)
**Glutamine**	1.24 (0.00)	1.23 (0.00)	99.10 (0.17)	98.17 (0.33)	1.24 (0.00)	1.24 (0.00)	99.08 (0.02)	98.59 (0.04)
**Glycine**	1.01 (0.23)	0.53 (0.39)	38.03 (8.62)	19.79 (14.78)	0.88 (0.36)	0.79 (0.25)	33.26 (13.71)	29.69 (9.39)
**Histidine**	0.07 (0.02)	0.05 (0.00)	10.96 (2.80)	7.92 (0.64)	0.07 (0.02)	0.05 (0.02)	10.31 (3.23)	7.33 (2.88)
**Isoleucine**	0.37 (0.05)	0.37 (0.05)	29.02 (3.59)	29.00 (3.80)	0.35 (0.08)	0.26 (0.06)	27.12 (6.30)	20.02 (4.45)
**Leucine**	0.57 (0.09)	0.33 (0.06)	17.64 (2.66)	10.19 (1.87)	0.60 (0.12)	0.43 (0.12)	18.60 (3.76)	13.42 (3.61)
**Lysine**	0.66 (0.03)	0.56 (0.06)	32.52 (1.36)	27.61 (2.88)	0.64 (0.04)	0.55 (0.08)	31.60 (2.15)	26.94 (3.68)
**Methionine**	0.03 (0.02)	-0.02 (0.02)	4.92 (3.55)	-3.68 (3.42)	0.04 (0.03)	0.00 (0.03)	6.50 (5.10)	0.29 (4.68)
**Phenylalanine**	0.24 (0.02)	0.24 (0.03)	17.02 (1.08)	16.99 (2.07)	0.25 (0.03)	0.18 (0.03)	17.76 (2.35)	13.09 (2.20)
**Serine**	2.76 (0.00)	2.71 (0.02)	98.24 (0.13)	96.77 (0.60)	2.70 (0.02)	2.72 (0.04)	96.23 (0.73)	96.89 (1.51)
**Threonine**	1.34 (0.01)	1.36 (0.02)	95.25 (0.75)	96.64 (1.23)	1.03 (0.07)	1.34 (0.02)	73.39 (4.99)	95.11 (1.64)
**Tryptophan**	-0.02 (0.02)	0.04 (0.02)	-6.40 (9.25)	14.23 (6.52)	-0.02 (0.02)	0.03 (0.01)	-6.73 (9.98)	12.03 (4.88)
**Tyrosine**	0.09 (0.01)	0.12 (0.02)	9.64 (1.51)	12.52 (1.61)	0.12 (0.03)	0.05 (0.05)	12.28 (2.66)	5.18 (5.17)
**Valine**	0.33 (0.07)	0.35 (0.06)	14.49 (3.29)	15.68 (2.64)	0.36 (0.19)	0.18 (0.12)	15.90 (8.26)	8.16 (5.21)

[a]Standard deviation is shown in parentheses
