# Supplementary material for: Effects of glucose availability in Lactobacillus sakei; metabolic change and regulation of the proteome and transcriptome
Source: PLoS One. 2017 Nov 3;12(11):e0187542. doi: 10.1371/journal.pone.0187542 (PMC5669474; doi:10.1371/journal.pone.0187542)
Supplement: S3 Table — (PDF) [file pone.0187542.s003.pdf]

S3 Table. Production of citrulline and ornithine in *L. sakei* during continuous cultivation in glucose-limited CDM-LAB medium at different growth rates.

	Strain 23K		Strain LS25	
	mM produced[a]		mM produced[a]	
	**0.357 h^{-1}**	**0.045 h^{-1}**	**0.357 h^{-1}**	**0.045 h^{-1}**
**Citrulline**	0.06 (0.00)	0.10 (0.01)	0.00 (0.00)	0.06 (0.03)
**Ornithine**	0.21 (0.00)	0.25 (0.02)	0.02 (0.01)	0.06 (0.03)

[a]Standard deviation is shown in parentheses
