# Supplementary figures and images for: Effects of glucose availability in Lactobacillus sakei; metabolic change and regulation of the proteome and transcriptome
Source: PLoS One. 2017 Nov 3;12(11):e0187542. doi: 10.1371/journal.pone.0187542 (PMC5669474; doi:10.1371/journal.pone.0187542)

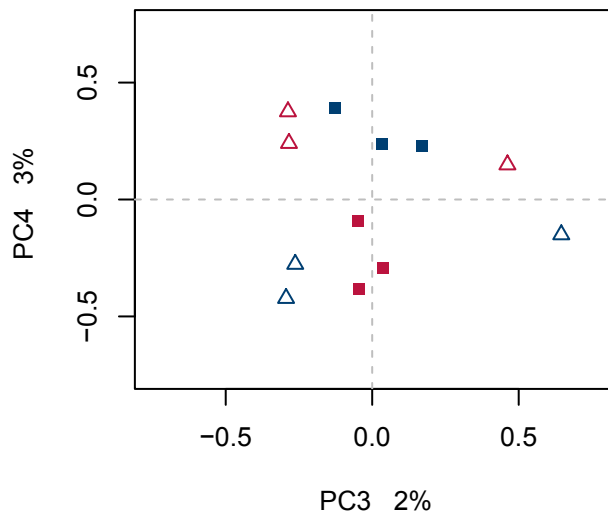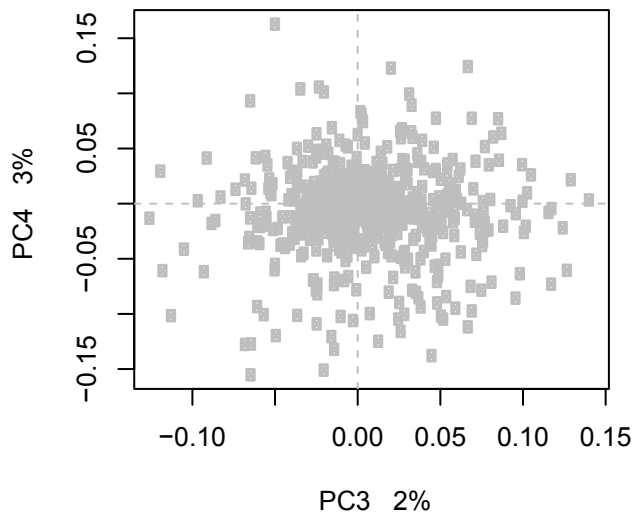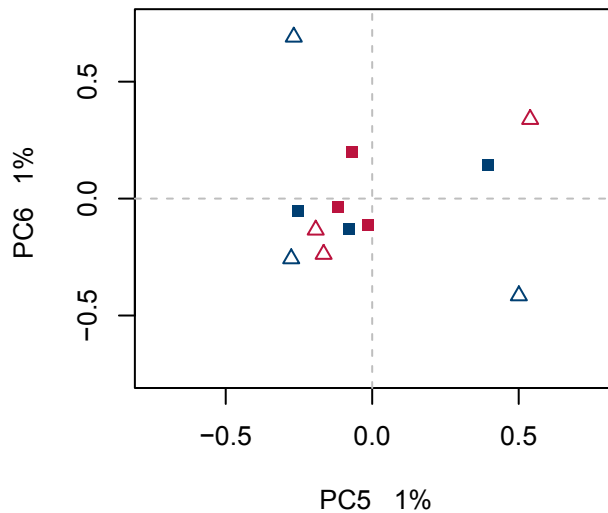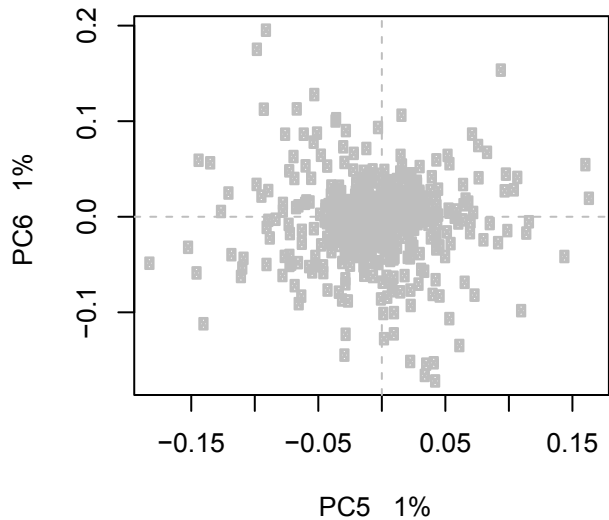

Supplement: S1 Fig — PCA of the proteome (all 643 variables) mean centered and standardized to unit variance. Score plots (left) and loading plots (right) on PC3 (x-axis) vs PC4 (y-axis) and PC5 (x-axis) vs PC6 (y-axis). L. sakei strains 23K and LS25 are shown in blue and red, respectively. Squares indicate high growth rate and open triangles indicate low growth rate. (PDF) [file pone.0187542.s005.pdf]

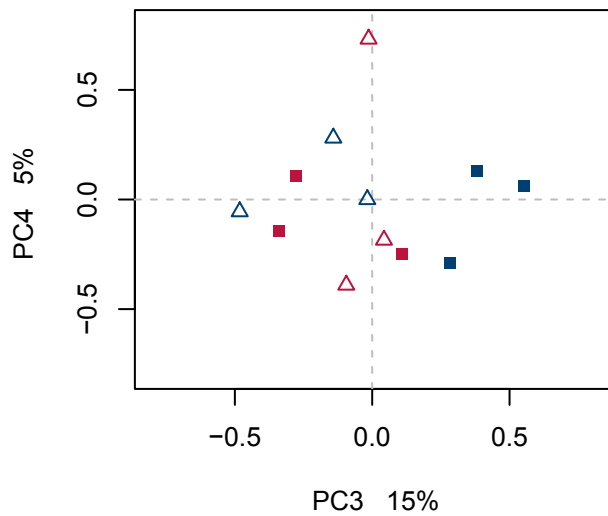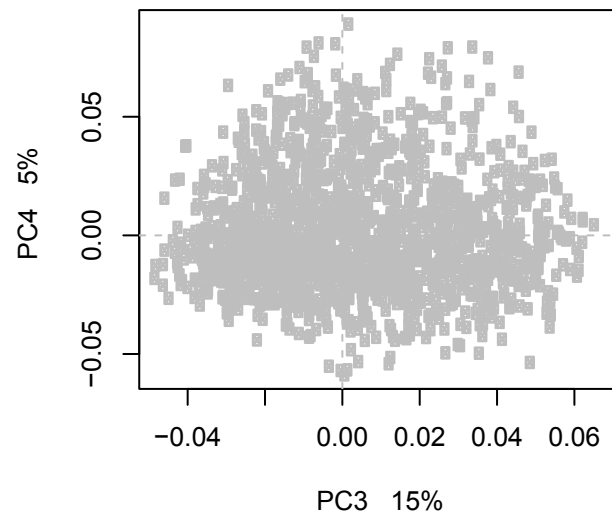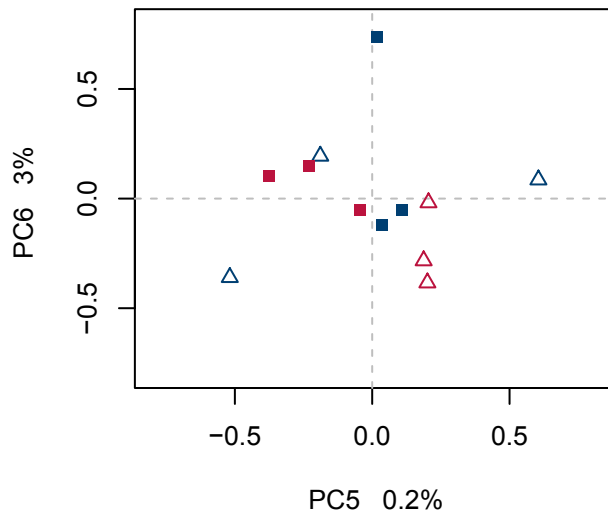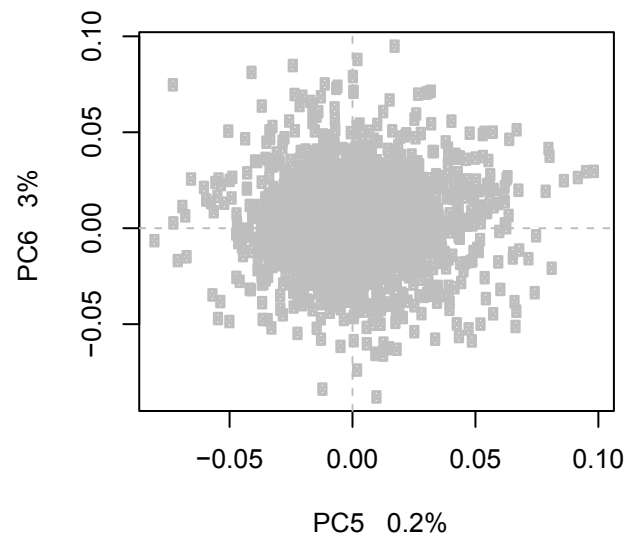

Supplement: S2 Fig — PCA of the transcriptome (all 1632 variables) mean centered and standardized to unit variance. Score plots (left) and loading plots (right) on PC3 (x-axis) vs PC4 (y-axis) and PC5 (x-axis) vs PC6 (y-axis). L. sakei strains 23K and LS25 are shown in blue and red, respectively. Squares indicate high growth rate and open triangles indicate low growth rate. (PDF) [file pone.0187542.s006.pdf]

# Proteome

# Transcriptome

Lactate

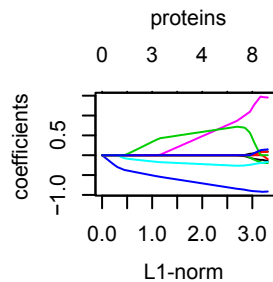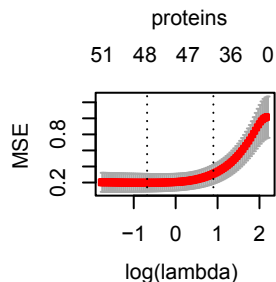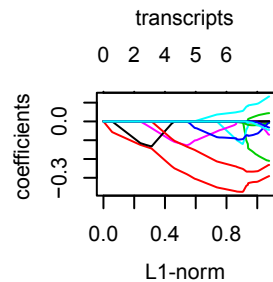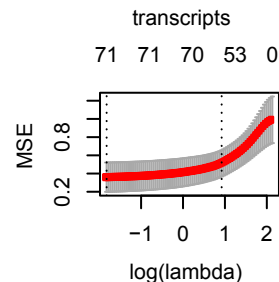

Formate

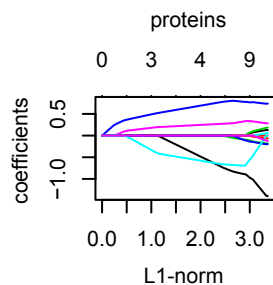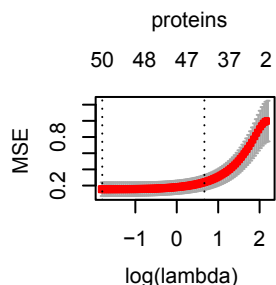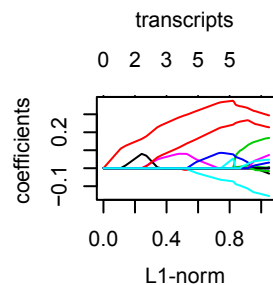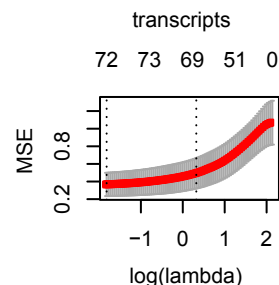

Acetate

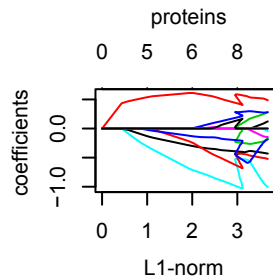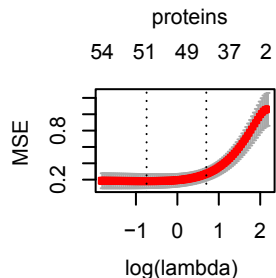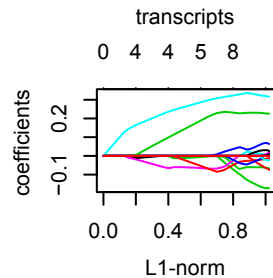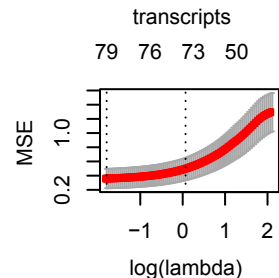

Ethanol

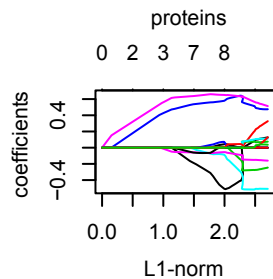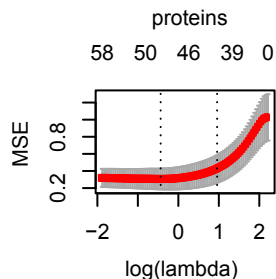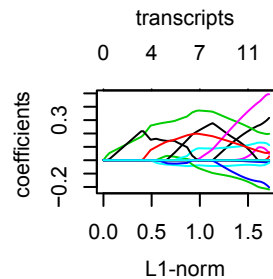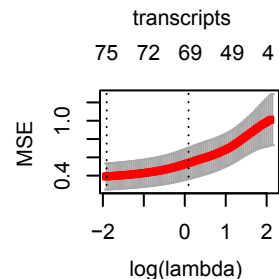

Supplement: S3 Fig — Coefficient curves (left plots) display the coefficients of each variable (the proteome or the transcriptome) in different colors with increasing number of variables in the model as the regularization parameter and thereby the L1-norm (sum of the absolute value of regression coefficients) changes. Cross-validation curves (right plots) are displayed in red, upper and lower standard deviation curves in grey, for the prediction of the phenome (lactate, formate, acetate and ethanol) based on input variables. MSE refers to Mean Square Error. (PDF) [file pone.0187542.s007.pdf]
