# Supplementary material for: Effects of glucose availability in Lactobacillus sakei; metabolic change and regulation of the proteome and transcriptome
Source: PLoS One. 2017 Nov 3;12(11):e0187542. doi: 10.1371/journal.pone.0187542 (PMC5669474; doi:10.1371/journal.pone.0187542)
Supplement: S4 Fig — The proteins (P) are listed by 23K locus tag (LCA_XXXX), protein name and group according to Table 2. Strains 23K and LS25 are shown in blue and red, respectively. Squares indicate high growth rate and open triangles indicate low growth rate. The proteins were selected by elastic net repeated 1000 times using alpha tuning parameter 0.5 and regularization parameter lambda set to log.lambda.min, followed by confidence intervals within each strain. (PDF) [file pone.0187542.s008.pdf]

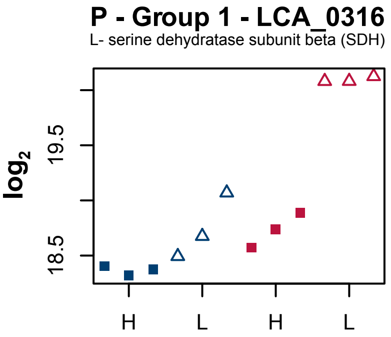
P - Group 1 - LCA_0316
L- serine dehydratase subunit beta (SDH)
log2
18.5
19.5
H
L
H
L

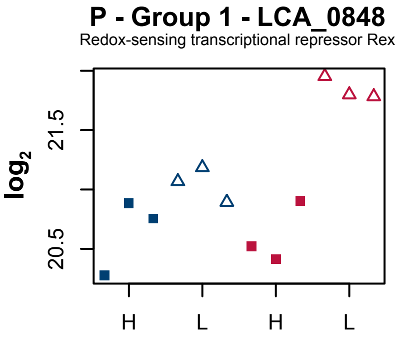
P - Group 1 - LCA_0848
Redox-sensing transcriptional repressor Rex
log2
20.5
21.5
H
L
H
L

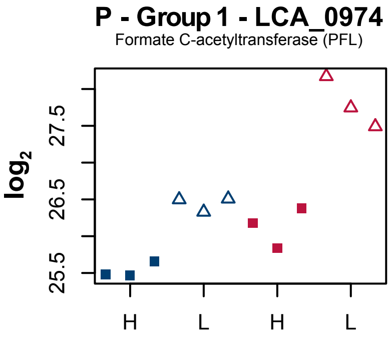
P - Group 1 - LCA_0974
Formate C-acetyltransferase (PFL)
log2
25.5
26.5
27.5
H
L
H
L

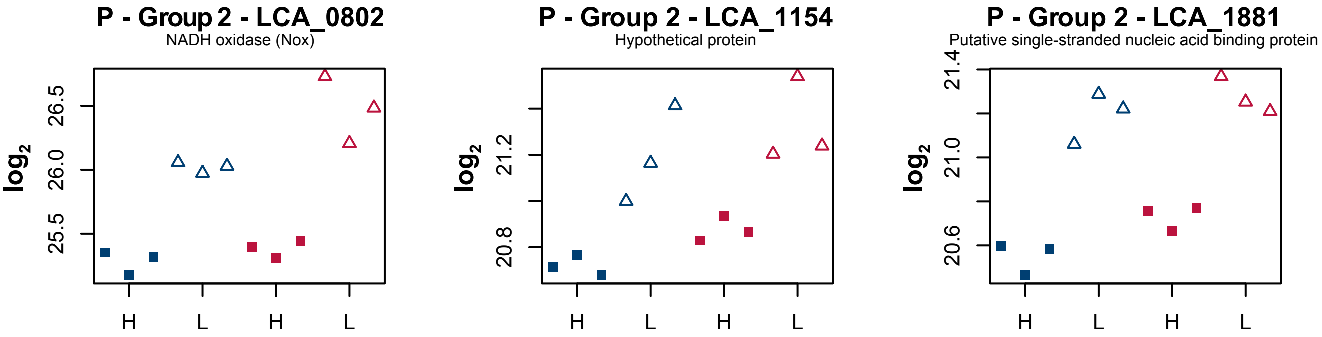
P - Group 2 - LCA_0802
NADH oxidase (Nox)
log2
25.5
26.0
26.5
H
L
H
L
P - Group 2 - LCA_1154
Hypothetical protein
log2
20.8
21.2
H
L
H
L
P - Group 2 - LCA_1881
Putative single-stranded nucleic acid binding protein
log2
20.6
21.0
21.4
H
L
H
L

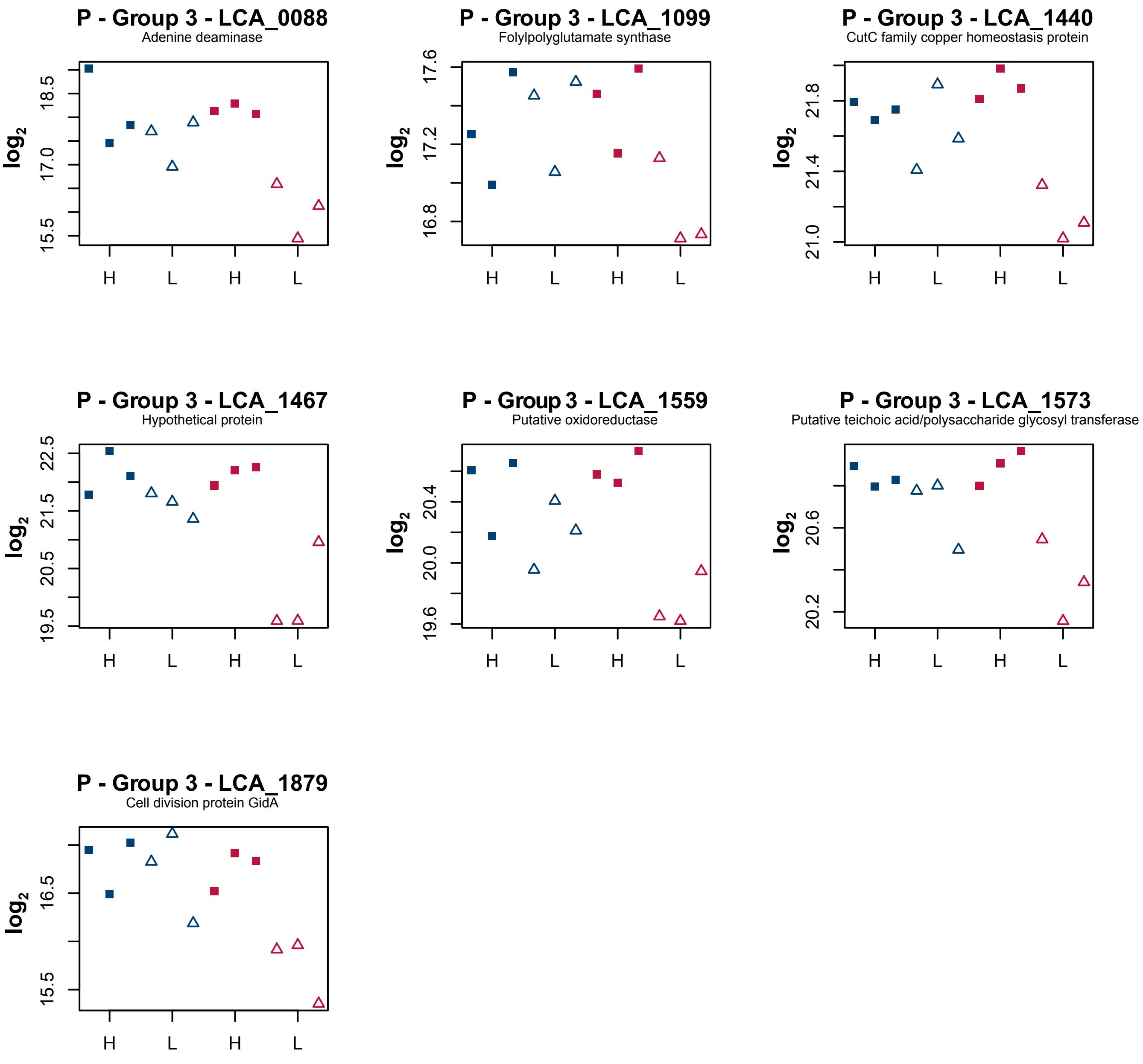
P - Group 3 - LCA_0088
Adenine deaminase
log2
15.5
17.0
18.5
H
L
H
L
P - Group 3 - LCA_1099
Folylpolyglutamate synthase
log2
16.8
17.2
17.6
H
L
H
L
P - Group 3 - LCA_1440
CutC family copper homeostasis protein
log2
21.0
21.4
21.8
H
L
H
L
P - Group 3 - LCA_1467
Hypothetical protein
log2
19.5
20.5
21.5
22.5
H
L
H
L
P - Group 3 - LCA_1559
Putative oxidoreductase
log2
19.6
20.0
20.4
H
L
H
L
P - Group 3 - LCA_1573
Putative teichoic acid/polysaccharide glycosyl transferase
log2
20.2
20.6
H
L
H
L
P - Group 3 - LCA_1879
Cell division protein GidA
log2
15.5
16.5
H
L
H
L

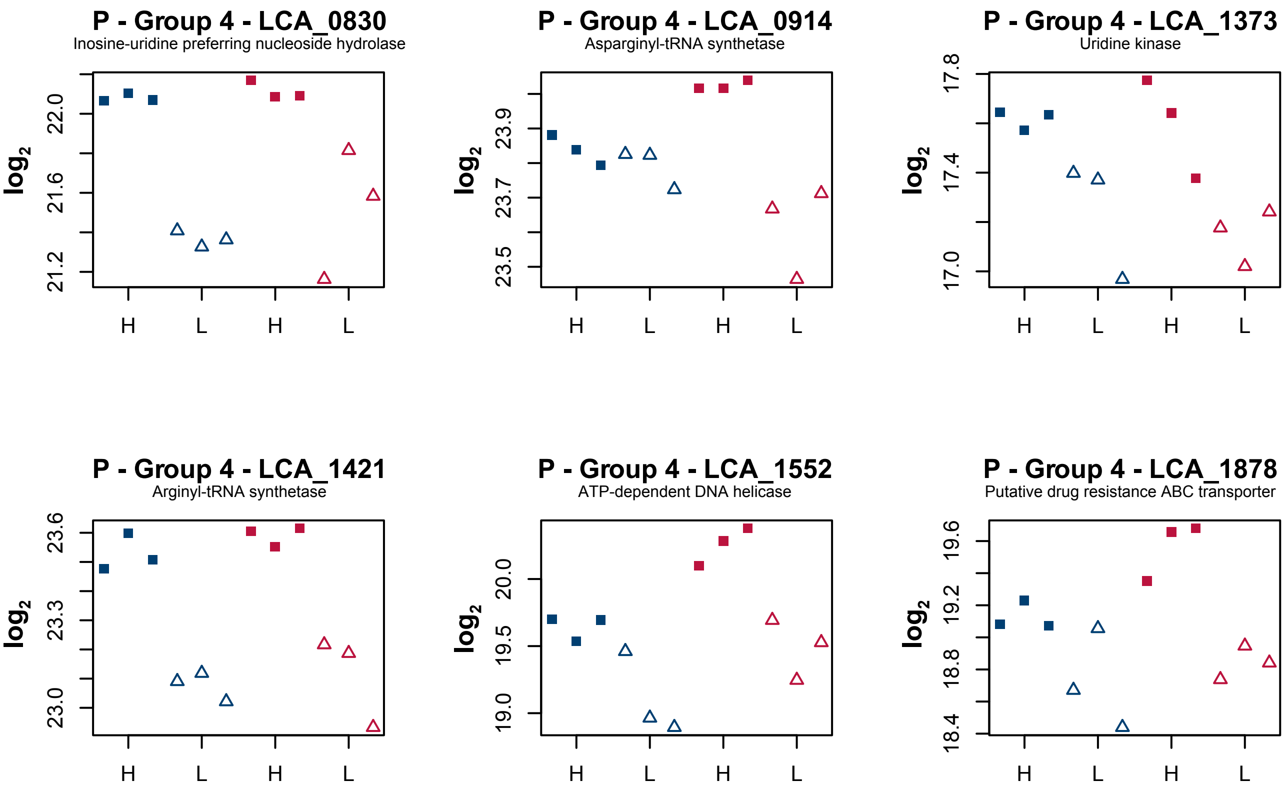
P - Group 4 - LCA_0830
Inosine-uridine preferring nucleoside hydrolase
log2
21.2
21.6
22.0
H
L
H
L
P - Group 4 - LCA_0914
Asparginyl-tRNA synthetase
log2
23.5
23.7
23.9
H
L
H
L
P - Group 4 - LCA_1373
Uridine kinase
log2
17.0
17.4
17.8
H
L
H
L
P - Group 4 - LCA_1421
Arginyl-tRNA synthetase
log2
23.0
23.3
23.6
H
L
H
L
P - Group 4 - LCA_1552
ATP-dependent DNA helicase
log2
19.0
19.5
20.0
H
L
H
L
P - Group 4 - LCA_1878
Putative drug resistance ABC transporter
log2
18.4
18.8
19.2
19.6
H
L
H
L
