# Supplementary material for: Effects of glucose availability in Lactobacillus sakei; metabolic change and regulation of the proteome and transcriptome
Source: PLoS One. 2017 Nov 3;12(11):e0187542. doi: 10.1371/journal.pone.0187542 (PMC5669474; doi:10.1371/journal.pone.0187542)
Supplement: S5 Fig — The gene transcripts (T) are listed by 23K locus tag (LCA_XXXX), gene product name and group according to Table 3. Strains 23K and LS25 are shown in blue and red, respectively. Squares indicate high growth rate and high glucose availability. Open triangles indicate low growth rate and restricted glucose availability. The gene transcripts were selected by elastic net repeated 1000 times using alpha tuning parameter 0.5 and regularization parameter lambda set to log.lambda.min, followed by confidence intervals within each strain. (PDF) [file pone.0187542.s009.pdf]

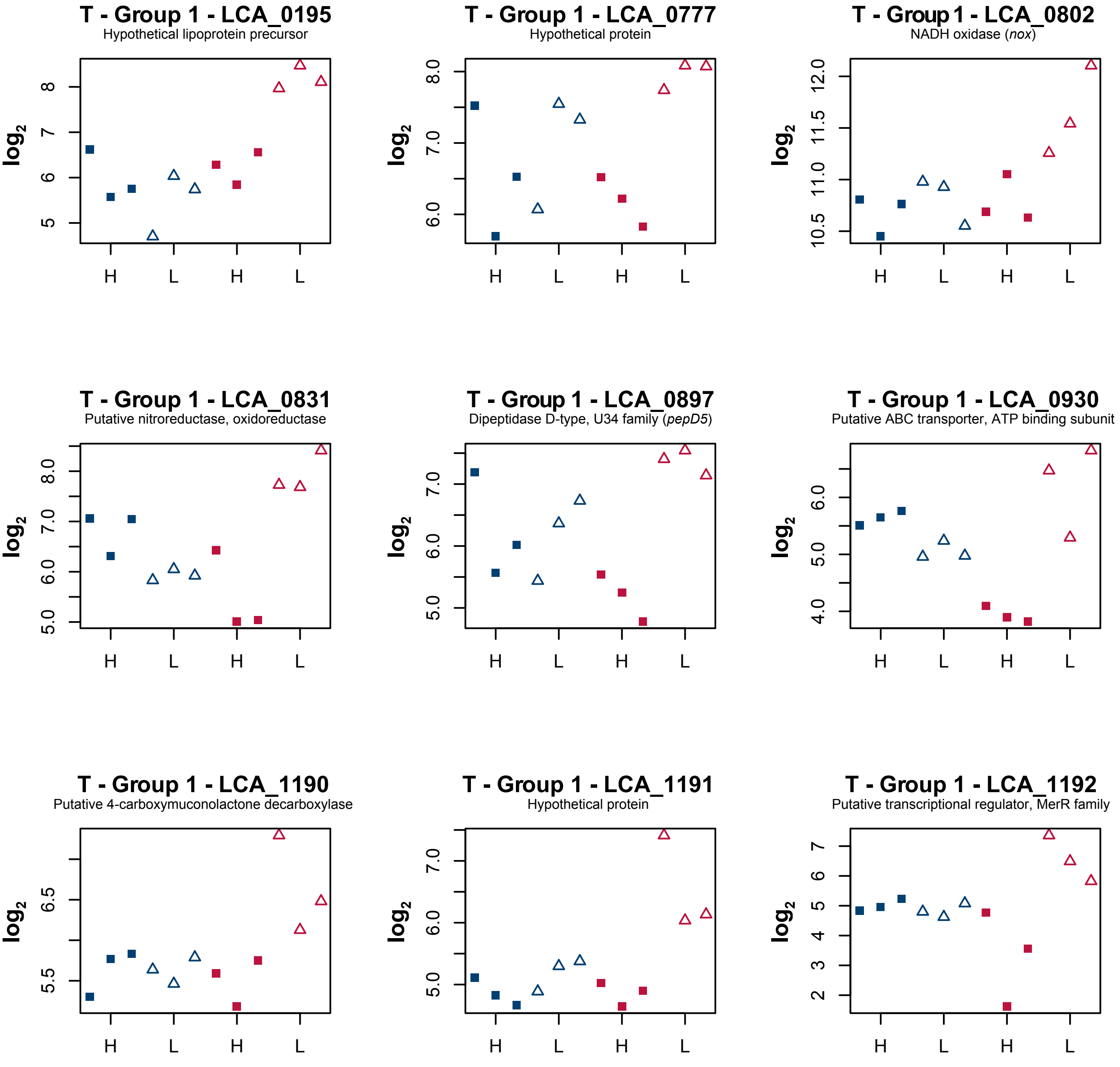
T - Group 1 - LCA_0195
Hypothetical lipoprotein precursor
T - Group 1 - LCA_0777
Hypothetical protein
T - Group 1 - LCA_0802
NADH oxidase (nox)
T - Group 1 - LCA_0831
Putative nitroreductase, oxidoreductase
T - Group 1 - LCA_0897
Dipeptidase D-type, U34 family (pepD5)
T - Group 1 - LCA_0930
Putative ABC transporter, ATP binding subunit
T - Group 1 - LCA_1190
Putative 4-carboxymuconolactone decarboxylase
T - Group 1 - LCA_1191
Hypothetical protein
T - Group 1 - LCA_1192
Putative transcriptional regulator, MerR family
log2
H L H L

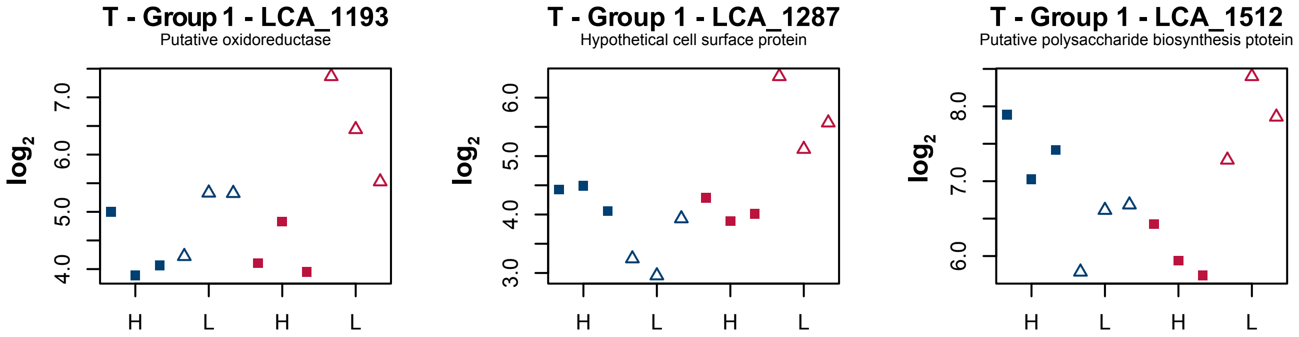

T - Group 1 - LCA_1193
Putative oxidoreductase
log2
4.0
5.0
6.0
7.0
H
L
H
L
T - Group 1 - LCA_1287
Hypothetical cell surface protein
log2
3.0
4.0
5.0
6.0
H
L
H
L
T - Group 1 - LCA_1512
Putative polysaccharide biosynthesis ptotein
log2
6.0
7.0
8.0
H
L
H
L

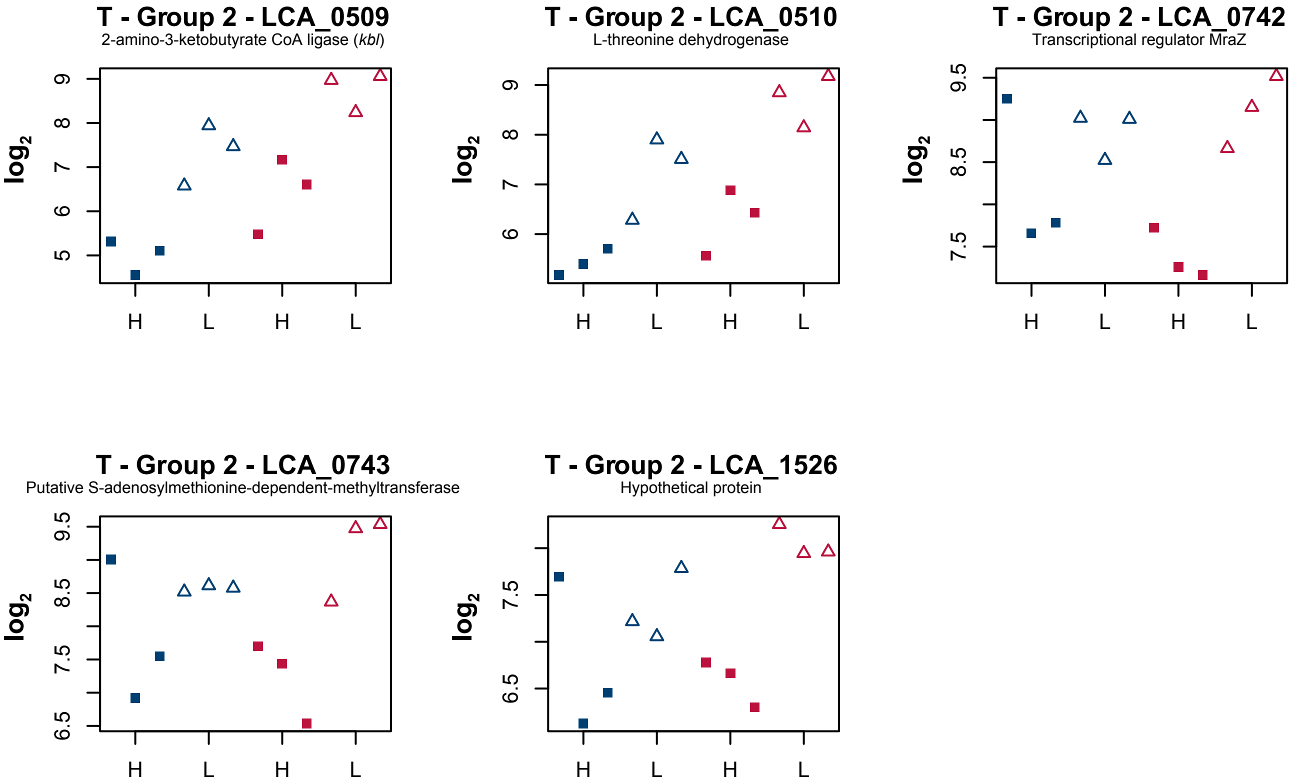

T - Group 2 - LCA_0509
2-amino-3-ketobutyrate CoA ligase (kbl)
T - Group 2 - LCA_0510
L-threonine dehydrogenase
T - Group 2 - LCA_0742
Transcriptional regulator MraZ
T - Group 2 - LCA_0743
Putative S-adenosylmethionine-dependent-methyltransferase
T - Group 2 - LCA_1526
Hypothetical protein
log2
H
L
H
L

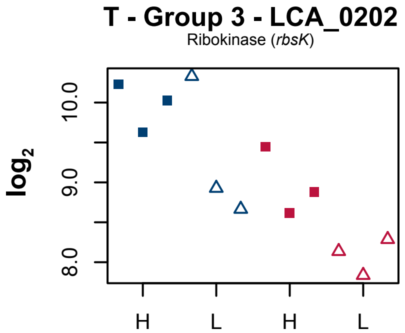
T - Group 3 - LCA_0202
Ribokinase (rbsK)
log2
10.0
9.0
8.0
H
L
H
L

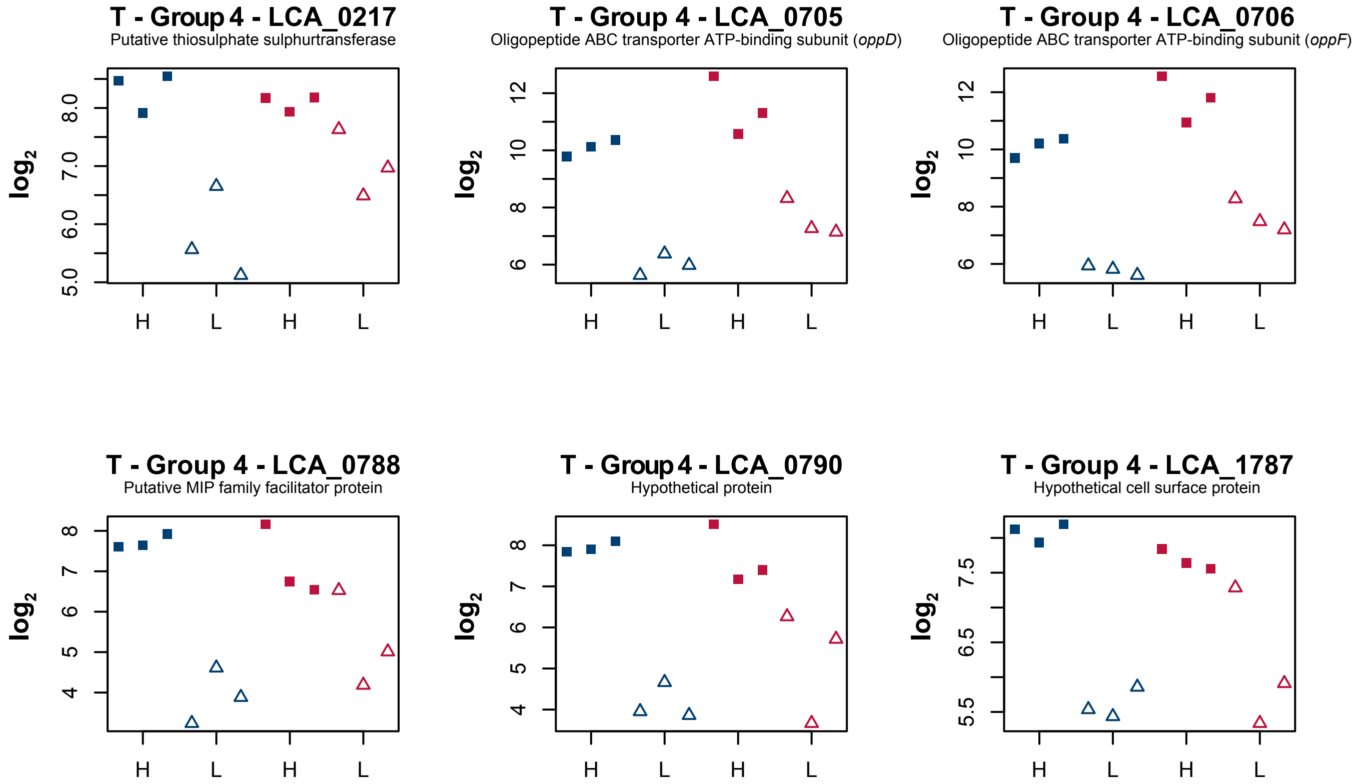
T - Group 4 - LCA_0217
Putative thiosulphate sulphurtransferase
T - Group 4 - LCA_0705
Oligopeptide ABC transporter ATP-binding subunit (*oppD*)
T - Group 4 - LCA_0706
Oligopeptide ABC transporter ATP-binding subunit (*oppF*)
T - Group 4 - LCA_0788
Putative MIP family facilitator protein
T - Group 4 - LCA_0790
Hypothetical protein
T - Group 4 - LCA_1787
Hypothetical cell surface protein
log_2
H
L
H
L
